# Multispectral image fusion for illumination-invariant palmprint recognition

**DOI:** 10.1371/journal.pone.0178432

**Published:** 2017-05-30

**Authors:** Longbin Lu, Xinman Zhang, Xuebin Xu, Dongpeng Shang

**Affiliations:** 1 MOE Key Lab for Intelligent Networks and Network Security, School of Electronics and Information Engineering, Xi’an Jiaotong University, Xi’an, China; 2 Guangdong Xi'an Jiaotong University Academy, Foshan, China; Nanjing Normal University, CHINA

## Abstract

Multispectral palmprint recognition has shown broad prospects for personal identification due to its high accuracy and great stability. In this paper, we develop a novel illumination-invariant multispectral palmprint recognition method. To combine the information from multiple spectral bands, an image-level fusion framework is completed based on a fast and adaptive bidimensional empirical mode decomposition (FABEMD) and a weighted Fisher criterion. The FABEMD technique decomposes the multispectral images into their bidimensional intrinsic mode functions (BIMFs), on which an illumination compensation operation is performed. The weighted Fisher criterion is to construct the fusion coefficients at the decomposition level, making the images be separated correctly in the fusion space. The image fusion framework has shown strong robustness against illumination variation. In addition, a tensor-based extreme learning machine (TELM) mechanism is presented for feature extraction and classification of two-dimensional (2D) images. In general, this method has fast learning speed and satisfying recognition accuracy. Comprehensive experiments conducted on the PolyU multispectral palmprint database illustrate that the proposed method can achieve favorable results. For the testing under ideal illumination, the recognition accuracy is as high as 99.93%, and the result is 99.50% when the lighting condition is unsatisfied.

## 1 Introduction

Nowadays, biometrics [1–3] plays an increasingly important role in the modern information society and has drawn more and more research attention throughout the world. As an emerging and promising biometric characteristic, palmprint possesses some remarkable advantages such as high distinguishability, excellent user-friendliness and strong stability. Generally speaking, palmprint recognition [4–7] is to verify the identity of a person based on the palm information including principal lines, wrinkles and fine ridges. In contrast to password cards or identification cards, palmprint recognition is much more convenient, efficient and reliable with extensive and successful applications [8]. However, it is still faced with some challenges in real noisy environments, where the illumination condition may be unsatisfied or even corrupted and then the performance of a palmprint recognition system based only on the visible spectrum degrades quickly. In addition, traditional methods obtain features from a single spectral band and consequently cannot achieve enough discriminative information of identities. In recent researches, there is a growing trend to use multispectral images instead of exploiting a single spectral image to improve the accuracy of a palmprint recognition system [9–12]. Images are captured at Blue, Green, Red and Near-infrared (NIR) spectral bands respectively, each of which commonly highlights different specific and complementary palm features. It is demonstrated that the utilization of multispectral images has made palmprint recognition as one of the most reliable and successful personal identification approaches.

Multispectral palmprint analysis is mainly focused on two separate directions, i.e., fusing multispectral information either at image level or at matching score level. For the first approach, the basis idea is to perform a multiscale decomposition on each source image, then integrate all these decompositions to form a composite representation, and finally reconstruct the fused image to be recognized by performing an inverse transform. Two major kinds of multiscale techniques, namely pyramid decomposition and wavelet decomposition, have been investigated in multispectral palmprint image fusion. A comparative research on multispectral palmprint image fusion was conducted in [13], where wavelet transform (WT), gradient pyramid (GP), morphological pyramid (MP) and curvelet transform (CT) were evaluated on two different spectral bands. Qualitative analysis demonstrated that the CT based image fusion could achieve a higher recognition accuracy. Besides, some other innovative methods, such as nonsubsampled contourlet transform (NSCT) [14, 15], discrete wavelet transform (DWT) [11, 12, 16], shift-invariant digital wavelet transform (SIDWT) [17, 18] and digital shearlet transform (DST) [19, 20], were widely and successfully used in multispectral palmprint image fusion. Alternatively, in the case of fusion at matching score level, palmprint features are extracted from different spectral bands separately, followed by a comparator to obtain a matching score. These matching scores in turn are fused using a sum rule and then verification is carried out on the fusion results. For example, Zhang et al. [21] employed the orientation-based coding (OC) for feature extraction of each spectral band and proposed a matching rule robust to the effect of information overlapping. In [22], Khan et al. applied the contour code (CC) for the representation of multispectral images before performing the matching score-level fusion. In [23], sum and weighted sum rules were utilized at the fusion stage. Additionally, some other explorations have been made in recent years. In [24], Hong et al. developed a novel hierarchical approach based on the block dominant orientation code (BDOC) and the block-based histogram of oriented gradient (BHOG) for feature-level fusion. Instead of using a fusion strategy, Xu et al. [25] presented a new method from a different perspective by utilizing the quaternion principal component analysis (QPCA) and the quaternion discrete wavelet transform (QDWT), which could fully extract the multispectral information.

Among the abovementioned works, the image fusion based scheme appears to be more attractive because it can effectively remove the noise that may be present during the acquisition process of palmprint images. Thus in this paper, we mainly concentrate on developing a novel method for illumination-invariant palmprint recognition by fusing multispectral information at image level. Firstly, the fast and adaptive bidimensional empirical mode decomposition (FABEMD) [26–28] is applied to each image captured at different spectral bands respectively, and then the fused image can be represented by the weighted sum of some bidimensional intrinsic mode functions (BIMFs). Secondly, a weighted Fisher criterion [29, 30] is introduced to select the proper fusion weights such that the fused image can contain enough discriminative information. Finally to improve the recognition accuracy and reduce the computation cost, a novel tensor-based extreme learning machine (TELM) [31–33] mechanism is proposed for classification of two-dimensional (2D) images. Extensive experiments under even or uneven illumination conditions are carried out on the PolyU multispectral palmprint database [11, 21, 34, 35] to show the superiority of our proposed method.

The rest of this paper is organized as follows: Section 2 introduces the multispectral imaging device and the region of interest (ROI) extraction method. Section 3 provides a schematic diagram of the proposed method and describes the FABEMD, the weighted Fisher criterion based image fusion strategy and the TELM in detail. Section 4 introduces the database and presents an experimental analysis. Finally, some concluding remarks are reported in Section 5.

## 2 Multispectral palmprint imaging and acquisition

**Fig 1:**
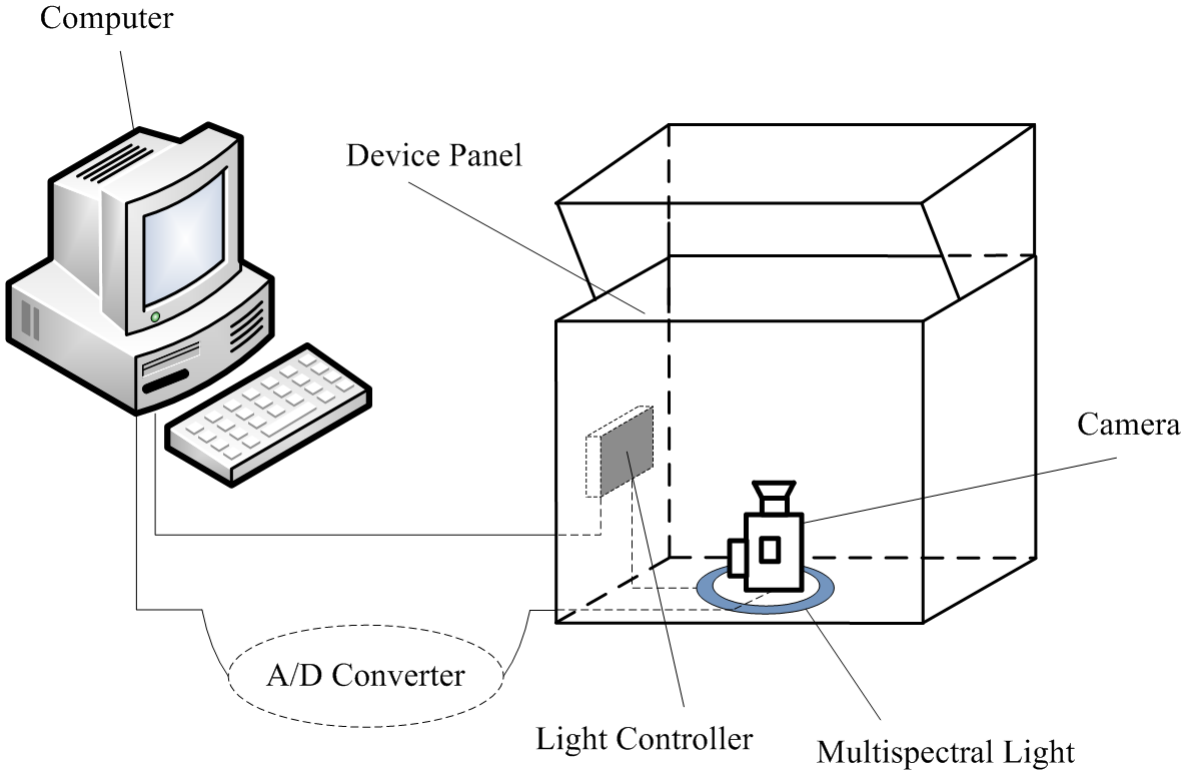
Structure of the multispectral palmprint imaging device.

**Fig 2:**
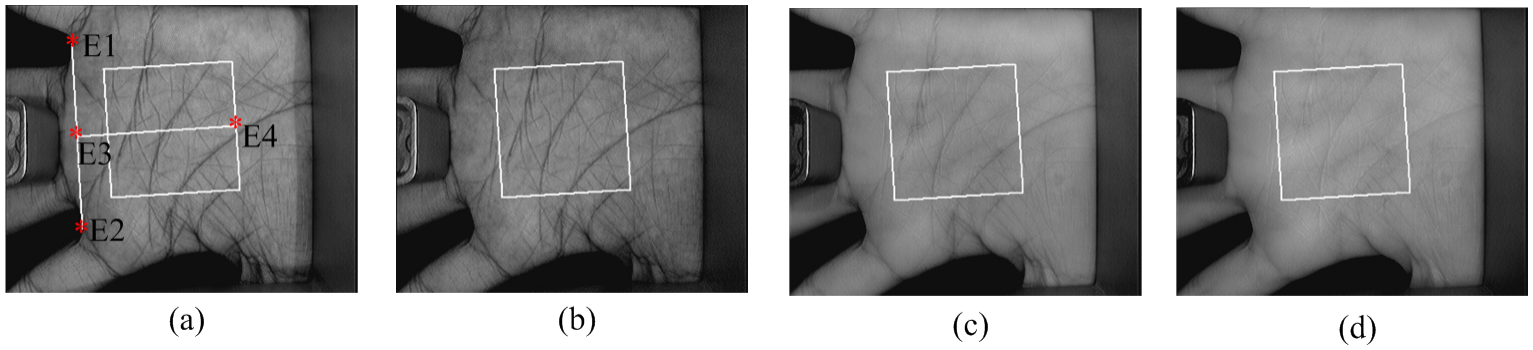
A typical multispectral palmprint sample: (a) Blue, (b) Green, (c) Red, and (d) NIR. The white square is the ROI of the image.

Before elaborating on the proposed method, we make an introduction to how the multispectral palmprint images are acquired and how the ROIs are located. Fig 1 shows the structure of the imaging device, which consists of a computer, an A/D converter, a CCD camera, a multispectral ring light source and a light controller. With signals from the light controller, the ring light source can successively generate four kinds of uniform illuminators at multiple spectral bands, i.e., Blue (470 nm), Green (525 nm), Red (660 nm) and NIR (880 nm). These four illuminators are switched between each other so quickly that a user’s multispectral palmprint images can be captured almost at the same time. Therefore the translation or rotation between two images is very small, making registration no longer necessary for image fusion. During the acquisition process, users are required to put their palms on the device panel where several pegs are employed to fix the placement of the hands. The CCD camera then acquires the palmprint images under the generated illuminators. Afterwards, by an A/D converter, the analog signals of images are converted to digital ones stored in the computer. Fig 2 illustrates a typical multispectral palmprint sample.

**Fig 3:**
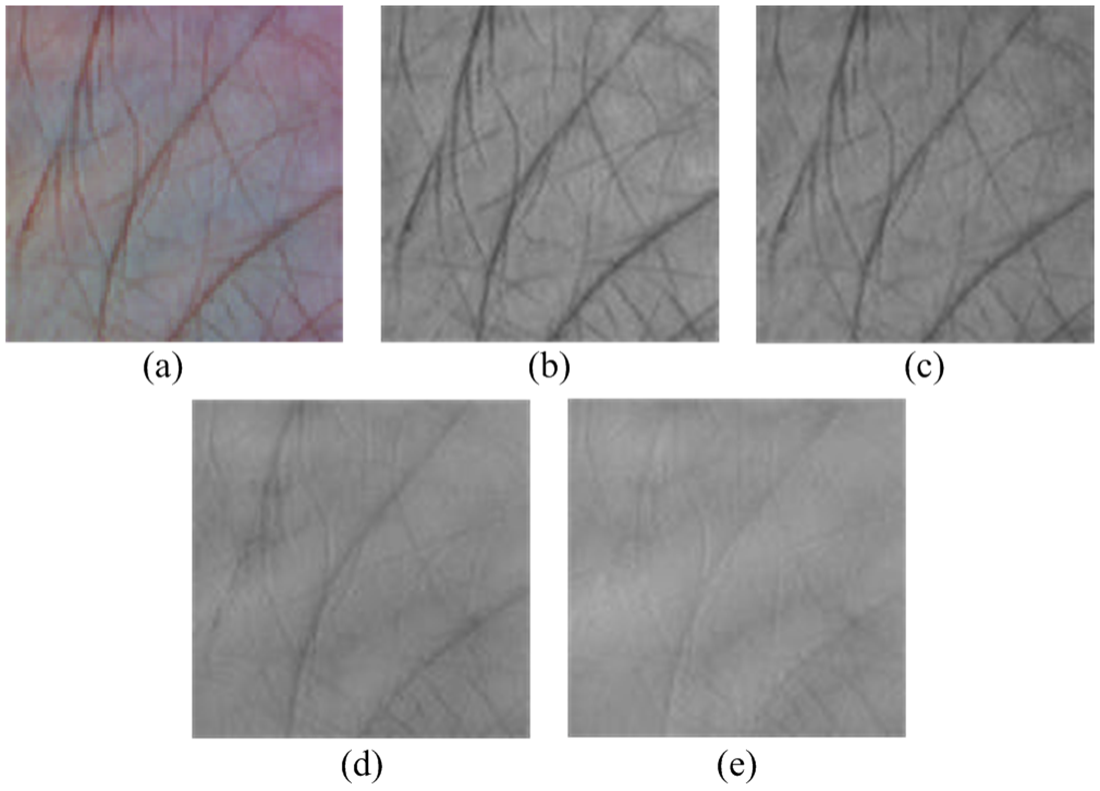
Extracted ROIs: (a) Color, (b) Blue, (c) Green, (d) Red, and (e) NIR.

Extracting an ROI from the acquired image is an essential step for multispectral palmprint recognition, which could efficiently decrease the effect of rotation and translation of the palm. As shown in Fig 2(a), by finding the two key points (E1, E2) located at the troughs between fingers, a coordinate system is built at the Blue band to crop the ROI. Here the line passing through E3 and E4 is the perpendicular bisector. Once the coordinate system is established, it is applied to the other spectral bands. The detailed steps are described in [34]. Fig 3 shows the extracted ROIs with a size of 128×128. Particularly, the palmprint images in color are also exhibited so that we can follow which channel is more informative visually.

## 3 Proposed multispectral palmprint image recognition method

**Fig 4:**
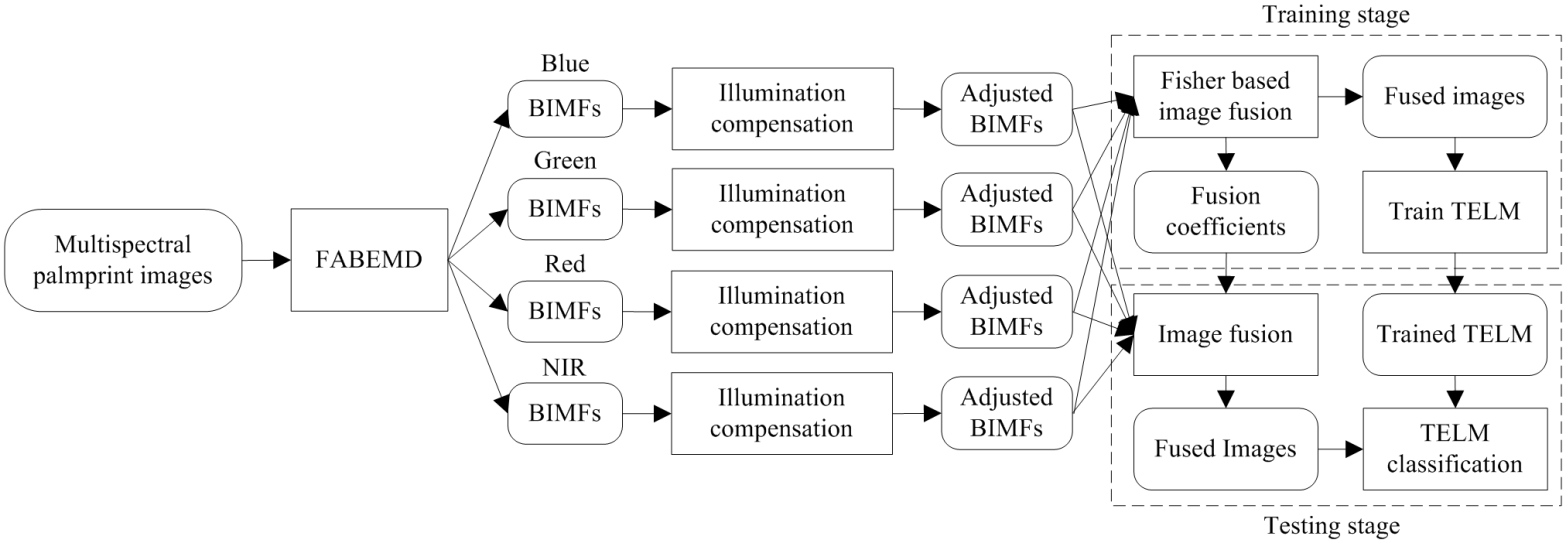
Outline of the proposed method.

Fig 4 illustrates the outline of the proposed palmprint recognition method, which mainly consists of three key steps: performing the FABEMD on the multispectral images and applying illumination compensation to the extracted BIMFs, determining the appropriate fusion coefficients based on the weighted Fisher criterion, and verifying the identities of palmprint images by a TELM classifier. Initially, for each spectral channel, the palmprint image is decomposed into some BIMFs and a residue using the FABEMD technique, where the residue can be considered as the estimation of the illumination condition at this spectral band. Based on the residue, BIMFs are adjusted with an illumination compensation operation. Afterwards, the fusion of multispectral images can be completed by calculating a weighted sum of all the adjusted BIMFs from Blue, Green, Red and NIR spectral bands. An improved Fisher criterion considering the neighborhood information is utilized to solve the fusion coefficients. By this means, the training samples in the fusion space can contain very discriminative information. In other words, the ratio of the between-class distance to the within-class distance in the fusion space tends to be maximized. Finally, the training fusion images are prepared in a tensor format and then employed to learn a TELM model. TELM combines tensor representation and extreme learning machine theory to determine the input weights and output weights of a single-hidden-layer feedforward neural network, by which the testing samples are classified.

### 3.1 Fast and adaptive bidimensional empirical mode decomposition

Fast and adaptive bidimensional empirical mode decomposition (FABEMD) is a data-driven signal analysis method that decomposes a 2D signal into its characteristic hierarchical components known as bidimensional intrinsic mode functions (BIMFs) [26]. It is based on an iterative shifting process, where the local extrema of the signals are initially detected and then the envelopes are estimated with the detected results. FABEMD adopts two kinds of order-statistics filters, namely MAX and MIN filters, to get the upper and lower envelopes, where the filter size is derived from the data.

Given a 2D signal **I**, FABEMD can represent it by

$$I=\sum_{i=1}^{K}S_{i}+R.$$ (1)

Here, *K* is the number of BIMFs decomposed from **I**, **S**_*i*_ denotes the *ith* BIMF, and **R** is the residue. In the shifting process, **S**_*i*_ is extracted from its source signal **J**_*i*_, where **J**_*i*_ = **J**_*i*−1_ − **S**_*i*−1_ and **J**_1_ = **I**. The detailed steps are explained as follows:

Step 1: Set *i* = 1 and initialize **J**_*i*_ = **I**.

**Fig 5:**
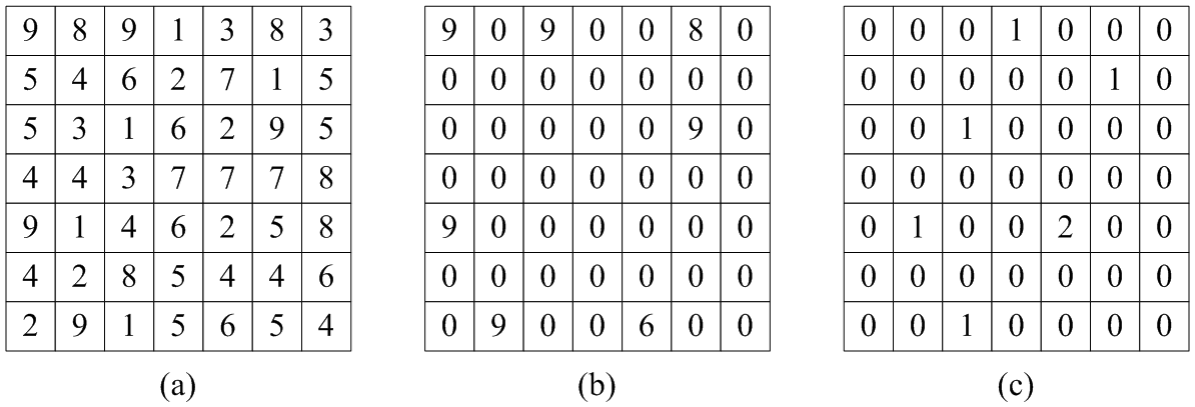
Demonstration of local maxima and minima maps: (a) source signal, (b) local maxima map, and (c) local minima map.

Step 2: Identify the local maxima and minima maps **M**_*i*_, **N**_*i*_ of **J**_*i*_.by exploiting a neighboring window search strategy as shown in Fig 5. A data point is regarded as the local extremum if its value is strictly higher or lower than all of the neighbors within the window. Particularly, for finding extrema points at the boundary or corner, the neighbors outside the image are neglected. Usually a window with a size of 3×3 is preferred for optimal results.

Step 3: Determine the proper window size for order-statistics filters based on the local maxima and minima maps **M**_*i*_, **N**_*i*_. For each local maximum point in **M**_*i*_, the Euclidean distance to the nearest other local maximum point is computed and stored in an adjacent maxima distance vector denoted as **d**_*adj*−max_. Similarly, an adjacent minima distance vector denoted as **d**_*adj*−max_ is calculated as well. The number of elements in the maxima (minima) adjacent vector is equal to the number of local maxima (minima) points. Considering a square window, the gross window size *w*_*en*−*g*_ for the order-statistics filters can be determined in two different ways as shown below:

$$\begin{array}{l} w_{en-g}=d_{1}\;=\text{ min}\left\{\text{min}\left\{d_{adj-\text{max}}\right\},\text{ min}\left\{d_{adj-\text{min}}\right\}\right\},\;or\\ w_{en-g}=d_{2}\;=\text{ max}\left\{\text{min}\left\{d_{adj-\text{max}}\right\},\text{ min}\left\{d_{adj-\text{min}}\right\}\right\}. \end{array}$$ (2)

The final window size *w*_*en*_ is obtained by rounding *w*_*en*−*g*_ to the nearest odd integer. Here, the range of *w*_*en*_ used for the multispectral palmprint images is from 3 to 69.

Step 4: Generate the upper and lower envelops **U**_*Ei*_, **L**_*Ei*_ by applying order-statistics and smoothing average filters. MAX and MIN filters with the window size of *w*_*en*_×*w*_*en*_ are employed to form the upper and lower envelops respectively according to the following equations:

$$\begin{array}{ll} U_{Ei}(m,n) & =\underset{(s,t)\in Z_{mn}}{\text{max}}J_{i}(s,t),\\ L_{Ei}(m,n) & =\;\underset{(s,t)\in Z_{mn}}{\text{min}}J_{i}(s,t), \end{array}$$ (3)

where the value **U**_*Ei*_(*m*, *n*) of the upper envelop at any point (*m*, *n*) is simply the maximum value of the elements in **J**_*i*_ in the region defined by *Z*_*mn*_. *Z*_*mn*_ is the square region with a size of *w*_*en*_×*w*_*en*_ centered at the point (*m*, *n*). Similarly the value **L**_*Ei*_(*m*, *n*) of the lower envelop at any point (*m*, *n*) is simply the minimum value of the elements in **J**_*i*_ in the region defined by *Z*_*mn*_. To attain smooth continuous surfaces for upper and lower envelopes, smoothing operations are performed on both **U**_*Ei*_(*m*, *n*) and **L**_*Ei*_(*m*, *n*), which may be stated as

$$\begin{array}{ll} U_{Ei}(m,n) & =\frac{1}{w_{en}\times w_{en}}\underset{(s,t)\in Z_{mn}}{\sum }U_{Ei}(s,t),\\ L_{Ei}(m,n) & =\frac{1}{w_{en}\times w_{en}}\underset{(s,t)\in Z_{mn}}{\sum }L_{Ei}(s,t). \end{array}$$ (4)

Step 5: Compute the *ith* BIMF by **S**_*i*_ = (**U**_*Ei*_ + **L**_*Ei*_)/2 and set *i*←*i* + 1, **J**_*i*_ = **J**_*i*−1_ − **S**_*i*−1_. Repeat steps 2 to 5 until the number of the extracted BIMFs is *K*.

**Fig 6:**
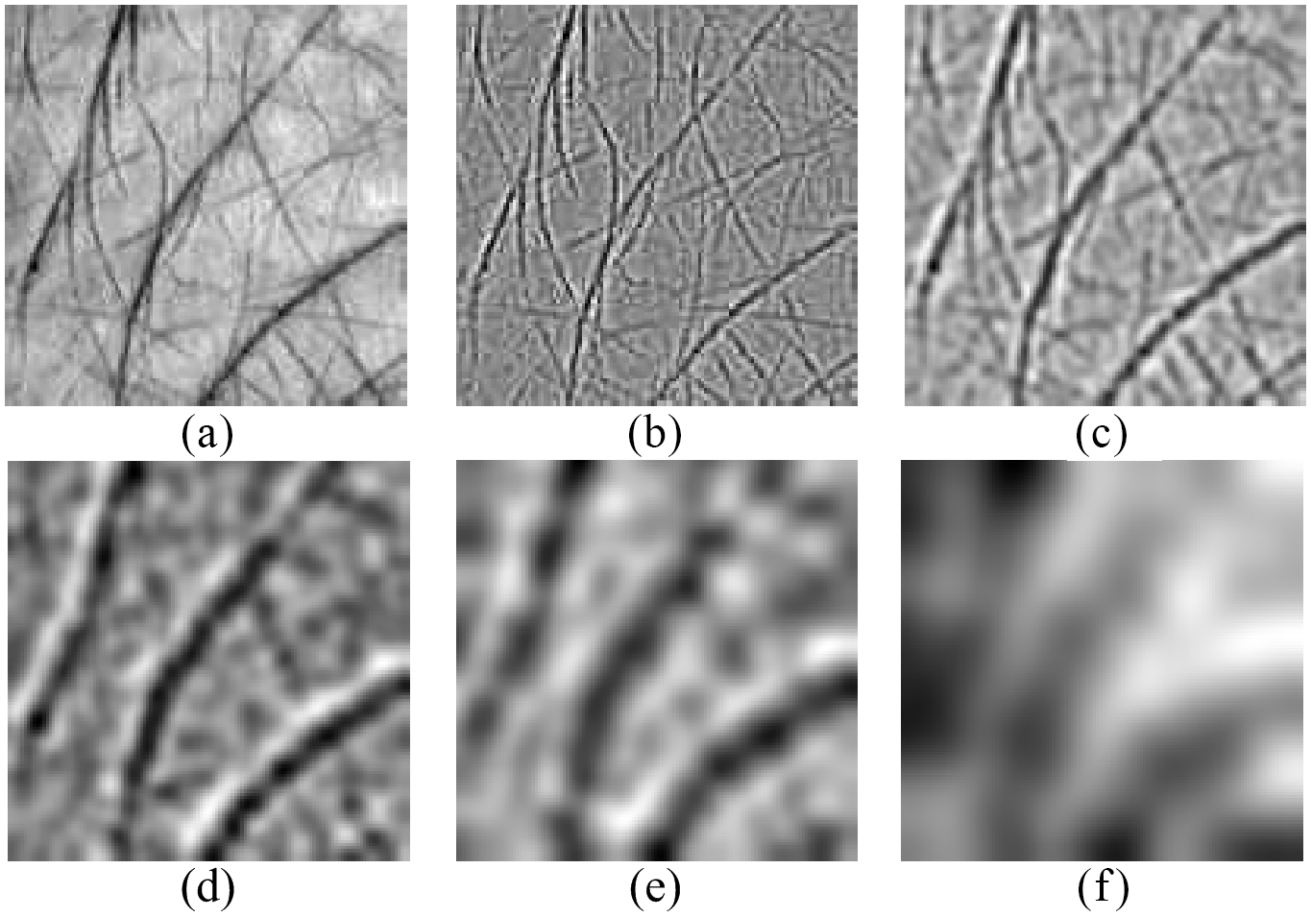
Decompositions of a palmprint image using FABEMD: (a) the source image, (b) the 1st BIMF, (c) the 2nd BIMF, (d) the 3rd BIMF, (e) the 4th BIMF, and (f) the residue.

Based on the above steps, a 2D signal is decomposed into *K* BIMFs **S**_*i*_, *i* = 1, …, *K*. Then the residue **R** can be calculated according to (Eq 1). The decomposition results of a palmprint image using FABEMD are shown in Fig 6.

**Fig 7:**
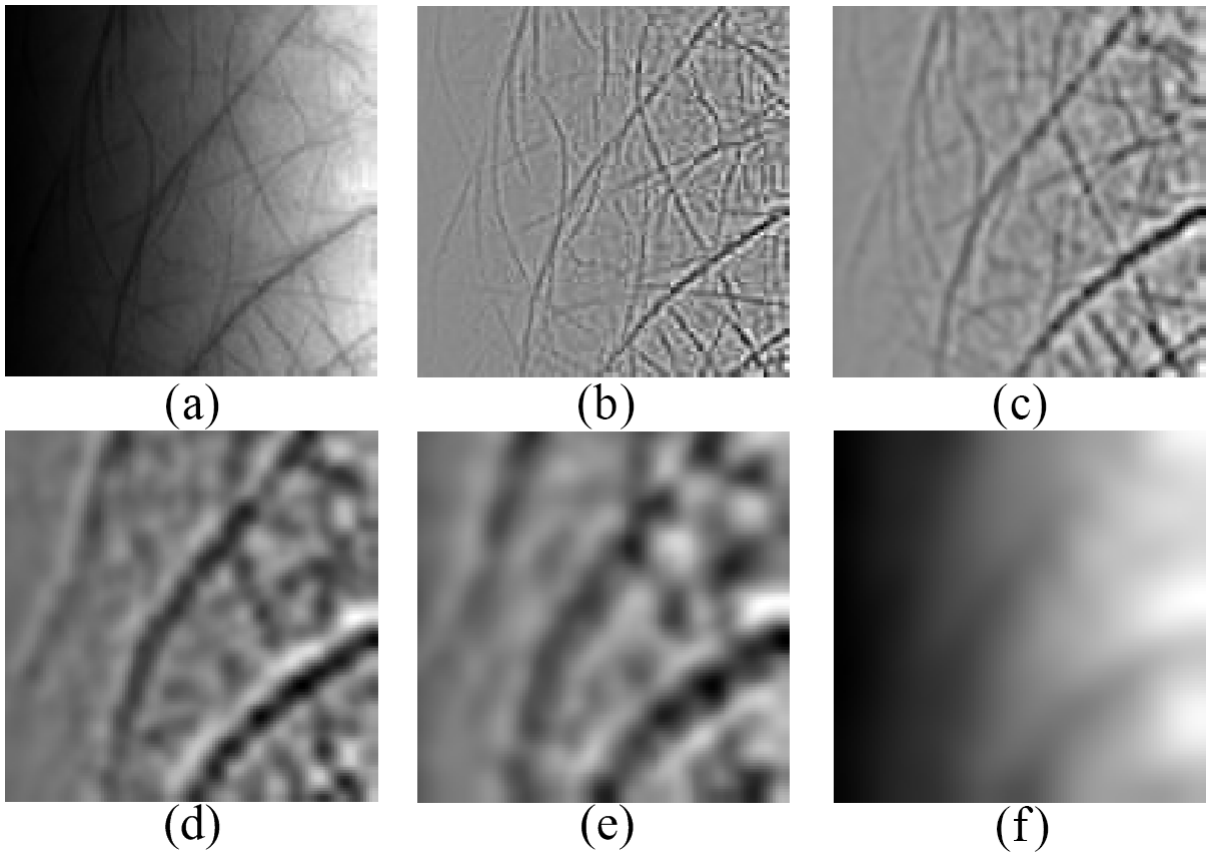
Decompositions of the noised image in Fig 6(a) using FABEMD: (a) the noised image, (b) the 1st BIMF, (c) the 2nd BIMF, (d) the 3rd BIMF, (e) the 4th BIMF, and (f) the residue.

In practical applications, the process of multispectral image acquisition is not so much restricted as described in Fig 1. For example, the images may be acquired in an open environment using a multispectral camera. The lighting condition is usually uncontrolled, and perhaps the images may be not uniformly illuminated. However, the results of FABEMD are very sensitive to the variation of lighting as demonstrated in Fig 7. In order to extract stable BIMFs, an illumination compensation method based on the residue of FABEMD is applied. Seen from Figs 6(f) and 7(f), the residue can be considered as a trend of the illumination. After an average filtering operation, the obtained smooth residue **R**_*s*_ is taken as the approximate illumination estimation. Then the adjusted BIMFs ${\tilde{S}}_{i}$ can be addressed by

$${\tilde{S}}_{i}(m,n)=\{\begin{array}{ll} S_{i}(m,n)/R_{s}(m,n), & if\;R_{s}(m,n)\neq 0\\ S_{i}(m,n)/eps, & otherwise \end{array},\;i=1,2,\cdots ,K$$ (5)

where *${\tilde{S}}_{i}(m,n),\;S_{i}(m,n)$* and **R**_*s*_ (*m*, *n*) are the values of ${\tilde{S}}_{i},\;S_{i}$ and **R**_*s*_ at any point (*m*, *n*), *eps* stands for a very small offset (In our paper, we set it as 1E-5).

**Fig 8:**
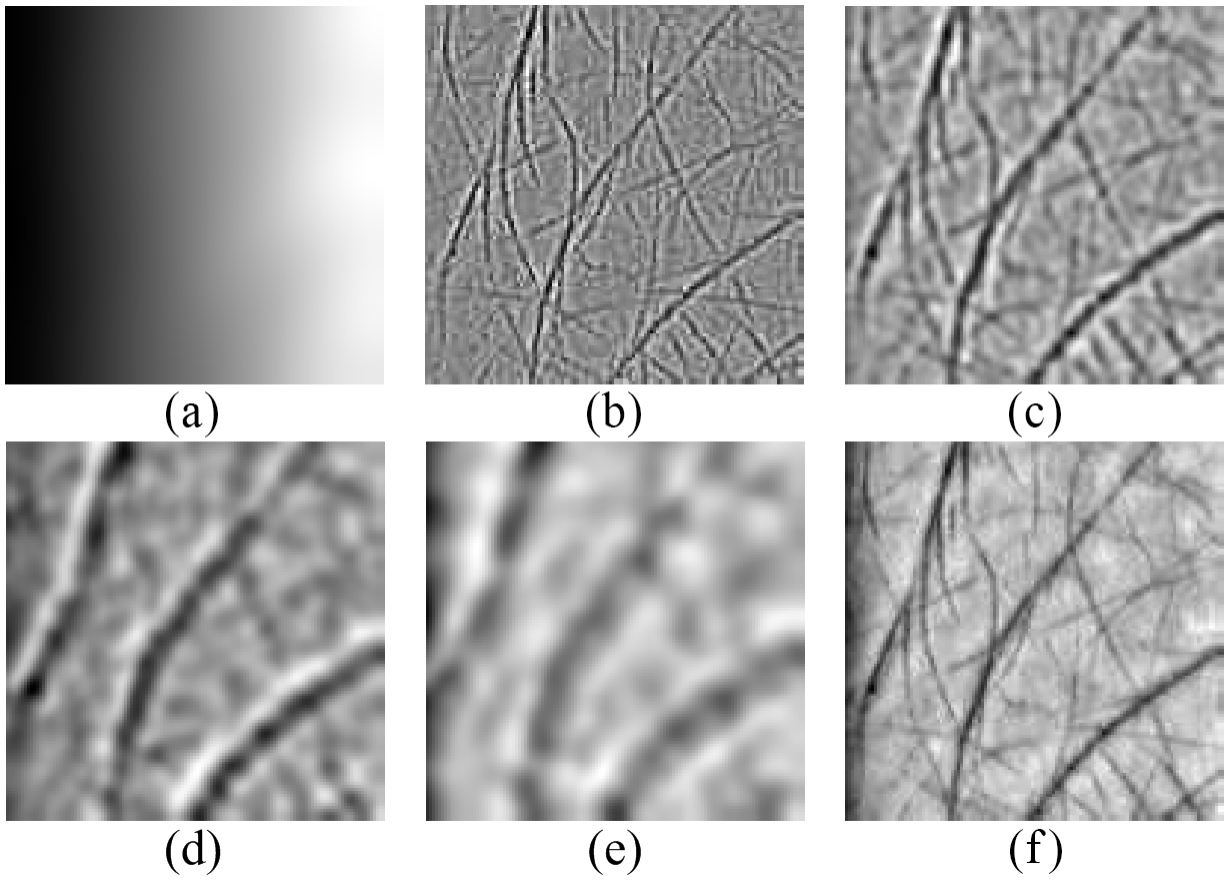
Demonstration of the adjusted results of Fig 7: (a) the smooth residue using an average filter with a size of 10×10, (b) the 1st BIMF, (c) the 2nd BIMF, (d) the 3rd BIMF, (e) the 4th BIMF, and (f) the reconstructed image by summing the K BIMFs.

Fig 8 shows the adjusted BIMFs. Compared with the decompositions exhibited in Fig 7, it can be seen that the uneven lighting condition is obviously improved by the illumination compensation operation. By this means, we can extract stable BIMFs which could be utilized to reconstruct the original image.

### 3.2 Image fusion based on the weighted Fisher criterion

Image fusion aims to combine the complementary information of multisource images and make the fused image more understandable and purposeful. For multispectral palmprint recognition [9–12], the task of image fusion is to reserve the useful features and remove the confusing identity information in each fusion component so that the images can be separated perfectly in the fusion space. For this purpose, an improved weighted Fisher criterion is applied to the BIMFs extracted from multispectral images.

For the *jth* palmprint sample, the corresponding vectorized BIMFs decomposed from Blue, Green, Red and NIR bands are denoted by $Vb_{i}^{j},Vg_{i}^{j},\;Vr_{i}^{j}$ and $Vn_{i}^{j}$ respectively. Here *j* = 1, 2, …, *N* and *i* = 1, 2, …, *K*. *N* is the number of palmprint samples. *K* is the number of BIMFs that an image is decomposed into.$Vb_{i}^{j}$, $Vg_{i}^{j}$, $Vr_{i}^{j}$ and $Vn_{i}^{j}$ are the *ith* adjusted BIMFs of the images captured at Blue, Green, Red and NIR bands of the *jth* palmprint sample. A general image fusion framework can be described by

$$F^{j}=\sum_{i=1}^{K}\left\{\varphi_{i}Vb_{i}^{j}+\varphi_{K+i}Vg_{i}^{j}+\varphi_{2*K+i}Vr_{i}^{j}+\varphi_{3*K+i}Vn_{i}^{j}\right\}=V^{j}\varphi ,$$ (6)

where $V^{j}=\left[Vb_{1}^{j},\cdots ,Vb_{K}^{j},\;Vg_{1}^{j},\cdots ,Vg_{K}^{j},Vr_{1}^{j},\cdots ,Vr_{K}^{j},Vn_{1}^{j},\cdots ,Vn_{K}^{j}\right]$ and **φ** = [φ_1_, φ_2_, …, φ_4*K_]^*T*^. Fusion based on the classic Fisher criterion [29] is to construct a set of fusion coefficients **φ** which could maximize the between-class distance and minimize the within-class distance simultaneously in the fusion space, that is

$$\begin{array}{ll} \varphi & =\underset{\varphi }{\text{argmax}}\frac{\sum_{j=1}^{N}{\left(V^{j}\varphi -\bar{V}\varphi \right)}^{T}(V^{j}\varphi -\bar{V}\varphi )-\sum_{l=1}^{m}\sum_{j=1}^{N_{l}}{\left(V_{\left(l\right)}^{j}\varphi -{\bar{V}}_{\left(l\right)}\varphi \right)}^{T}(V_{\left(l\right)}^{j}\varphi -{\bar{V}}_{\left(l\right)}\varphi )}{\sum_{l=1}^{m}\sum_{j=1}^{N_{l}}{\left(V_{\left(l\right)}^{j}\varphi -{\bar{V}}_{\left(l\right)}\varphi \right)}^{T}(V_{\left(l\right)}^{j}\varphi -{\bar{V}}_{\left(l\right)}\varphi )}\\ & =\;\underset{\varphi }{\text{argmax}}\frac{\sum_{j=1}^{N}{\left(V^{j}\varphi -\bar{V}\varphi \right)}^{T}(V^{j}\varphi -\bar{V}\varphi )}{\sum_{l=1}^{m}\sum_{j=1}^{N_{l}}{\left(V_{\left(l\right)}^{j}\varphi -{\bar{V}}_{\left(l\right)}\varphi \right)}^{T}(V_{\left(l\right)}^{j}\varphi -{\bar{V}}_{\left(l\right)}\varphi )}\\ & =\underset{\varphi }{\text{argmax}}\frac{\varphi^{T}\sum_{j=1}^{N}{\left(V^{j}-\bar{V}\right)}^{T}(V^{j}-\bar{V})\varphi }{\varphi^{T}\sum_{l=1}^{m}\sum_{j=1}^{N_{l}}{\left(V_{\left(l\right)}^{j}-{\bar{V}}_{\left(l\right)}\right)}^{T}(V_{\left(l\right)}^{j}-{\bar{V}}_{\left(l\right)})\varphi }\\ & =\underset{\varphi }{\text{argmax}}\frac{\varphi^{T}D\varphi }{\varphi^{T}D_{w}\varphi } \end{array},$$ (7)

$$\begin{array}{ll} D & =\sum_{j=1}^{N}{\left(V^{j}-\bar{V}\right)}^{T}(V^{j}-\bar{V}),\\ D_{w} & =\sum_{l=1}^{m}\sum_{j=1}^{N_{l}}{\left(V_{\left(l\right)}^{j}-{\bar{V}}_{\left(l\right)}\right)}^{T}(V_{\left(l\right)}^{j}-{\bar{V}}_{\left(l\right)}), \end{array}$$ (8)

where $\bar{V}$ is the mean of all samples **V**^*j*^, *j* = 1, 2, …, *N*, ${\bar{V}}_{\left(l\right)}$ is the mean of the samples belonging to the *lth* class (Here, class means the identity of the palmprint), $V_{\left(l\right)}^{j}$ is the *jth* sample of the *lth* class, *N*_*l*_ is the number of samples of the *lth* class, *m* is the number of classes and $N=\sum_{l=1}^{m}N_{l}$. Then the fusion coefficient vector **φ** is obtained by solving the generalized eigenvalue decomposition:

$$D\varphi =\lambda D_{w}\varphi .$$ (9)

Here, **φ** is the eigenvector corresponding to the largest eigenvalue.

A drawback of the traditional Fisher criterion is that it pays equal attention to every sample when constructing the fusion coefficient vector. In fact, the samples near the class center maintain relative rest in the projection from decomposition space to fusion subspace. Meanwhile, the samples close to the border should be projected towards their corresponding class centers and keep farther away from other class points. In other words, the closer to the class center these samples are, the less contribution they make to the projection. Whereas, the farther away from the class center and the closer to the border those samples are, the more contribution they make. Inspired by this fact, a contribution factor μ^*j*^ of a sample **V**^*j*^ is defined as

$$\mu^{j}=\frac{\sum_{i,V^{i}\in \Psi_{b}^{j}}^{}\text{exp}(-{\left\|V^{i}-V^{j}\right\|}^{2}/\delta^{2})}{\sum_{i,V^{i}\in \Psi^{j}}^{}\text{exp}(-{\left\|V^{i}-V^{j}\right\|}^{2}/\delta^{2})},$$ (10)

where Ψ^*j*^ is the set of the k-nearest neighbors of the *jth* sample **V**^*j*^, $\Psi_{b}^{j}$ is the subset of Ψ^*j*^ with classes different from the one of **V**^*j*^, and δ is the spread of Gaussian. From this definition, it can be inferred that when a sample is located inside the class with no between-class samples surrounded, the contribution factor μ^*j*^ is zero. When a sample is near the border and its k-nearest neighbors are not all from the same class, the value of μ^*j*^ is nonzero. Moreover, when the number of between-class samples increases and the distance of between-class samples decreases, the value of μ^*j*^ becomes larger and the contribution of this sample is greater. An extreme condition is that the value of μ^*j*^ is one, meaning that all the k-nearest neighbors are from other classes.

Based on the contribution factor, a weighted Fisher criterion is proposed. A large weight is arranged to the sample located close to the border and a small weight is given to the sample near the class center:

$$\begin{array}{ll} \varphi & =\underset{\varphi }{\text{argmax}}\frac{\varphi^{T}\sum_{j=1}^{N}\mu^{j}{\left(V^{j}-\bar{V}\right)}^{T}(V^{j}-\bar{V})\varphi }{\varphi^{T}\sum_{l=1}^{m}\sum_{j=1}^{N_{l}}\mu_{\left(l\right)}^{j}{\left(V_{\left(l\right)}^{j}-{\bar{V}}_{\left(l\right)}\right)}^{T}(V_{\left(l\right)}^{j}-{\bar{V}}_{\left(l\right)})\varphi }\\ & =\underset{\varphi }{\text{argmax}}\frac{\varphi^{T}D\varphi }{\varphi^{T}D_{w}\varphi } \end{array},$$ (11)

where $\mu_{\left(l\right)}^{j}$ is the contribution factor of the *jth* sample of the *lth* class, **D** and **D**_*w*_ are redefined as

$$\begin{array}{ll} D & =\sum_{j=1}^{N}\mu^{j}{\left(V^{j}-\bar{V}\right)}^{T}(V^{j}-\bar{V}),\\ D_{w} & =\sum_{l=1}^{m}\sum_{j=1}^{N_{l}}\mu_{\left(l\right)}^{j}{\left(V_{\left(l\right)}^{j}-{\bar{V}}_{\left(l\right)}\right)}^{T}(V_{\left(l\right)}^{j}-{\bar{V}}_{\left(l\right)}). \end{array}$$ (12)

**Fig 9:**
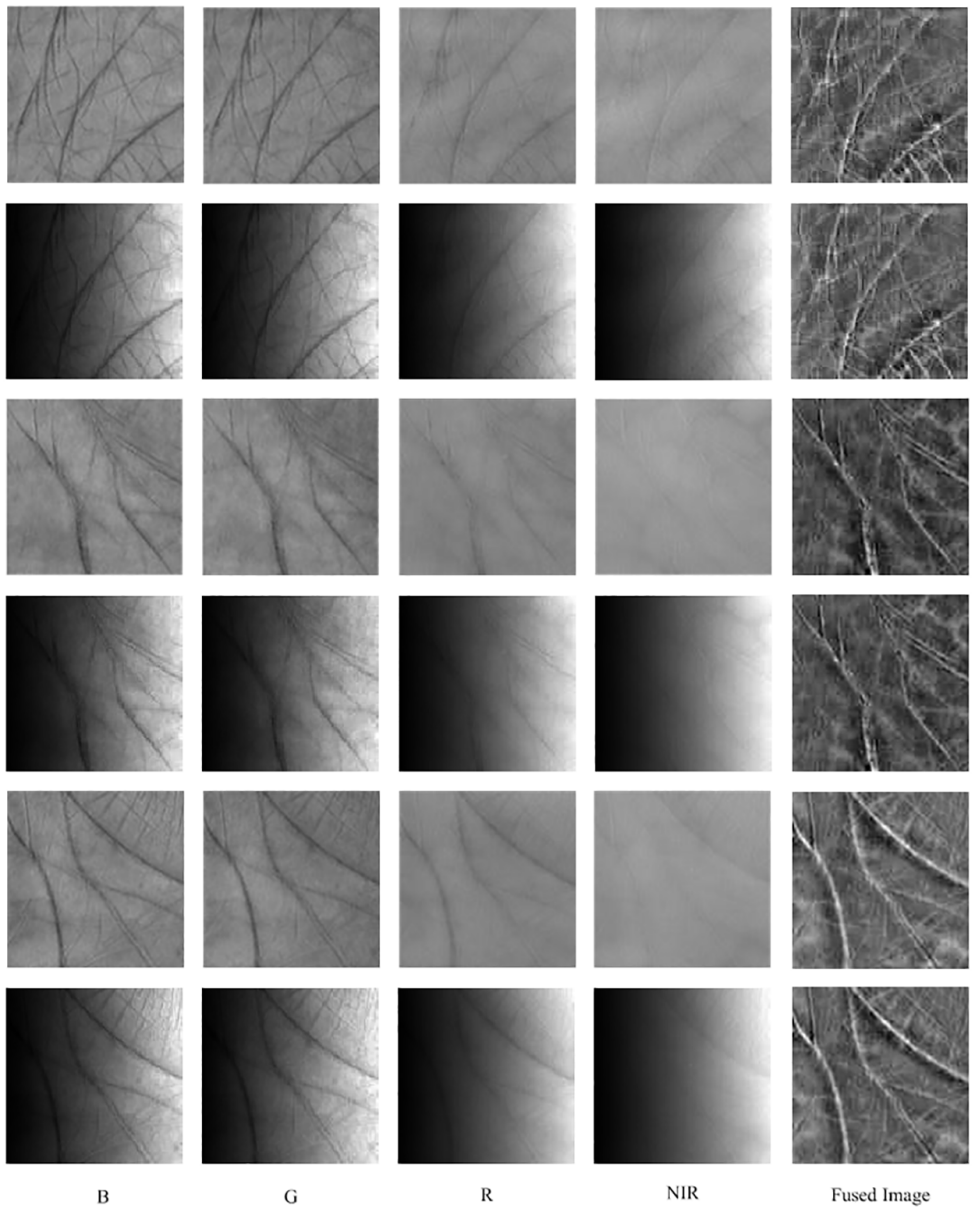
Demonstration of the weighted Fisher criterion based image fusion under different illumination conditions. Each row illustrates a multispectral palmprint sample and the corresponding fusion image.

The fusion vector **φ** can be computed with a generalized eigenvalue decomposition according to (Eq 9), and then the fused image is achieved by (Eq 6). Fig 9 shows the weighted Fisher criterion based image fusion results under different illumination conditions. As expected, the fused images include all the detailed information from each spectral band. Moreover, we can find that the fused images are nearly not influenced by the lighting change.

### 3.3 Tensor-based extreme learning machine

Extreme learning machine (ELM) is a novel training method for single-hidden-layer feedforward neural networks (SLFNs) with the hidden nodes randomly assigned and then fixed without iteratively tuning [32]. It has gained comprehensive interest due to its fast learning speed, good generalization ability and ease of implementation. However, ELM is originally proposed for one-order tensor (i.e. vector) classification. In the case of higher-order signals, they must be preliminarily vectorized, which may lose some structure information and degrade the final classification performance. In our work, we have made an improvement of the traditional ELM based on tensor decomposition. All input training samples are represented by a higher-order tensor. The input weights of an SLFN are calculated by applying a higher-order singular value decomposition (HOSVD) technique [31], and then the output weights are analytically determined by the simple generalized inverse operation.

For *N* distinct samples {**x**_*i*_, **t**_*i*_}, *i* = 1, 2, …, *N*, **x**_*i*_ is a 1×*n* input vector and **t**_*i*_ is a 1×*m* output vector. In our work, **x**_*i*_ represents the vectorized fused image of the *ith* sample and *n* denotes the number of pixels in the fused image. **t**_*i*_ is the class label and *m* denotes the number of classes. Training an SLFN with $\tilde{N}$ hidden nodes is to find the suitable input weights $\alpha_{j},\;j=1,2,\cdots ,\tilde{N}$ and output weights $\beta_{j},\;j=1,2,\cdots ,\tilde{N}$ such that

$$f_{\tilde{N}}(x_{i})=\sum_{j=1}^{\tilde{N}}g(x_{i}^{e}\alpha_{j})\beta_{j}=t_{i},\;i=1,2,\cdots ,N,$$ (13)

where **α**_*j*_ is a (*n* + 1)×1 vector and denotes the weight vector connecting the input nodes to the *jth* hidden node, **β**_*j*_ is a 1×*m* vector and denotes the weight vector connecting the *jth* hidden node to the output nodes, $x_{i}^{e}$ is the augmenting vector of **x**_*i*_ with the format of [**x**_*i*_ 1] ∈ *R*^*n*+1^, and *g*(*x*) is the activation function (e.g., sigmoid and threshold). Here, we select sigmoid $g(x)=\frac{1}{1+e^{-x}}$ as the activation function. The above formula can be written compactly as

Hβ=T, (14)

where $\beta ={\left[\begin{array}{c} \beta_{1}\\ \vdots \\ \beta_{\tilde{N}} \end{array}\right]}_{\tilde{N}\times m}$, $T={\left[\begin{array}{c} t_{1}\\ \vdots \\ t_{N} \end{array}\right]}_{N\times m}$ and $H={\left[\begin{array}{ccc} g(x_{1}^{e}\alpha_{1}) & \cdots & g(x_{1}^{e}\alpha_{\tilde{N}})\\ \vdots & \cdots & \vdots \\ g(x_{N}^{e}\alpha_{1}) & \cdots & g(x_{N}^{e}\alpha_{\tilde{N}}) \end{array}\right]}_{N\times \tilde{N}}$. Furthermore, **H** is described in a more compact way as

$$H=g(x^{e}\alpha ),$$ (15)

where $x^{e}={\left[\begin{array}{c} x_{1}^{e}\\ \vdots \\ x_{N}^{e} \end{array}\right]}_{N\times (n+1)}$ and $\alpha ={\left[\begin{array}{ccc} \alpha_{1} & \cdots & \alpha_{\tilde{N}} \end{array}\right]}_{(n+1)\times \tilde{N}}$.

The ELM theory has proved that, if the activation functions are infinitely differentiable, the hidden layer output matrix **H** can be obtained by using a random map **α** with (Eq 15). Afterwards, the output weight matrix **β** is calculated by

$$\beta =H^{†}T,$$ (16)

where **H**^†^ is the Moore—Penrose generalized inverse of **H**.

Different from ELM designed only for signals in vector format, our proposed tensor-based ELM (TELM) is an extension for higher-order signals. Here, tensors are the generalization of vectors with orders higher than one. A tensor $A\in R^{\left(I_{1}\times I_{2}\times \cdots \times I_{p}\right)}$ has order *p*. *I*_1_, *I*_2_, …, *I*_*p*_ represent the number of elements for each dimension. Instead of using a random map, a HOSVD-based method is employed in TELM to construct the multidimensional feature projection matrices, by which the input training data are mapped into the hidden layer.

Firstly, we introduce two basic operations in HOSVD. The matrix unfolding $A^{\left(q\right)}\in R^{Iq\times \prod_{i\neq q}I_{i}}$ of a tensor $A\in R^{\left(I_{1}\times I_{2}\times \cdots \times I_{p}\right)}$ along dimension *q* is defined as

$$\begin{array}{l} A\Rightarrow_{q}A^{\left(q\right)},\\ A_{}^{\left(q\right)}(i_{q},j)=A(i_{1},\cdots ,iq,\cdots ,i_{p}),\;j=1+\overset{p}{\underset{l=1,l\neq q}{\sum }}(i_{l}-1)\overset{p}{\underset{o=l+1,o\neq q}{\prod }}I_{o}. \end{array}$$ (17)

The product between a tensor $A\in R^{\left(I_{1}\times I_{2}\times \cdots \times I_{q}\times \cdots \times I_{p}\right)}$ and a matrix $B\in R^{J_{q}\times I_{q}}$ is denoted by

$$C=A\times_{q}B,$$ (18)

where $C\in R^{I_{1}\times I_{2}\times \cdots \times J_{q}\times \cdots \times I_{p}}$ is a tensor with the elements computed by

$$C(i_{1},i_{2},\cdots ,j_{q},\cdots ,i_{p})=\sum_{i_{q}}A(i_{1},i_{2},\cdots ,i_{q},\cdots ,i_{p})B(j_{q},i_{q}).$$ (19)

Note that the matrix unfolding **C**^(*q*)^ along dimension *q* is the product between **B** and **A**^(*q*)^, that is

$$C^{\left(q\right)}=BA^{\left(q\right)}.$$ (20)

Given *N* distinct training samples in higher-order format $\{x_{i}\in R^{I_{1}\times I_{2}\times \cdots \times I_{p}},t_{i}\in R^{m}\},\;i=1,2,\cdots ,N$, where **x**_*i*_ is the 2D fused image in our work and $x_{i}\in R^{I_{1}\times I_{2}},\;I_{1}=I_{2}=128$, the core task for TELM is to construct the multidimensional projection matrices. For this purpose, we first prepare the input training tensor as

$$\begin{array}{l} \Gamma \in R^{N\times I_{1}\times I_{2}},\\ \Gamma (i,i_{1},i_{2})=x_{i}(i_{1},i_{2}),\;i=1,2,\cdots ,N,\;i_{1}=1,2,\cdots ,I_{1},\;i_{2}=1,2,\cdots ,I_{2}. \end{array}$$ (21)

Then the HOSVD decomposes the training tensor **Γ** as

$$\Gamma =Z\times_{2}U_{2}\times_{3}U_{3},$$ (22)

where **U**_2_, **U**_3_ are the multidimensional projection matrices and **Z** is the hidden layer input tensor. For *i* = 2, 3, **U**_*i*_ can be computed from the standard SVD of the unfolding matrix Γ^(*i*)^, i.e.,$\Gamma^{\left(i\right)}=U_{i}\Sigma_{i}V_{i}^{T}$. **U**_*i*_ is the orthogonal matrix that contains the orthonormal vectors spanning the column space of the matrix unfolding **Γ**^(*i*)^. Then the tensor **Z** can be addressed by using the inversion formula:

$$Z=\Gamma \times_{2}U_{2}^{T}\times_{3}U_{3}^{T}.$$ (23)

Actually, we use the simple truncation of the first ${\tilde{N}}_{1},{\tilde{N}}_{2}$ columns of the matrices **U**_2_, **U**_3_ to calculate the hidden layer output matrix **H**, that is

$$\begin{array}{ll} Z & =\Gamma \times_{2}U_{2,{\tilde{N}}_{1}}^{T}\times U_{3,{\tilde{N}}_{2}}^{T},\\ H & =g(Z^{\left(1\right)}). \end{array}$$ (24)

This truncation operation can not only maintain the discriminative multidimensional projections but also greatly reduce the computational cost. With the multidimensional projection matrices, the input tensors are mapped into the feature subspace. Finally, we can achieve the output weight matrix **β** of the hidden layer through (Eq 16).

In the TELM algorithm, the multidimensional feature projection matrices are utilized as the input weights, which effectively reserves the structure information of the input tensors. The output weights are calculated by solving the generalized inverse. There are no iterative learning steps and thus the learning speed is very fast.

## 4 Experiments

In this section, we report the experimental results and evaluate the performance of the proposed method. The recognition accuracy (*RA*) indicator is used as the assessment standard and it is defined as

$$RA=\frac{Num_{c}}{Num},$$ (25)

where *Num*_*c*_ stands for the number of correctly recognized samples and *Num* is the total number of testing samples.

All the experiments were conducted on a machine with a 2.50 GHz Intel core^™^ processor and 8 GB memory. MATLAB 2012a was used as the simulation software.

### 4.1 Multispectral palmprint database

**Fig 10:**
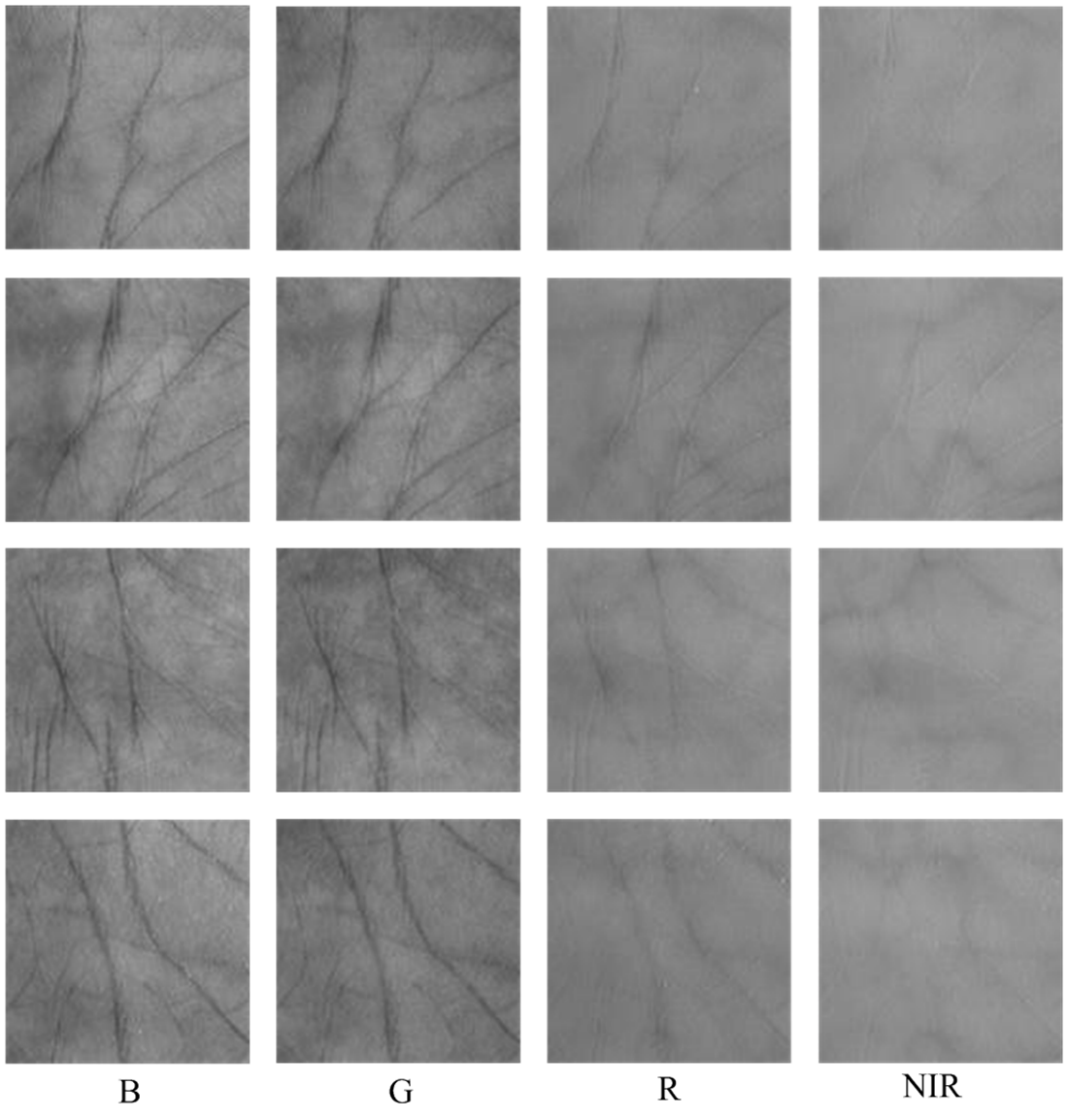
Demonstration of multispectral palmprint images in the PolyU database. Each row shows a multispectral palmprint sample.

We conducted the experiments on the PolyU multispectral palmprint database offered by Hong Kong Polytechnic University [11, 21, 34, 35]. All the images were collected from 250 volunteers (195 males and 55 females) aged from 20 to 60 years old. The acquisition was completed in two different sessions, each lasting about 9 days. In one session, the subject was required to provide 6 images for his left and right palms respectively. The palmprint images were acquired at four spectral bands, i.e., Red, Green, Blue and NIR. For each band, there are 6,000 images obtained from 500 different palms in total. Fig 10 shows some multispectral palmprint samples in the PolyU database.

**Fig 11:**
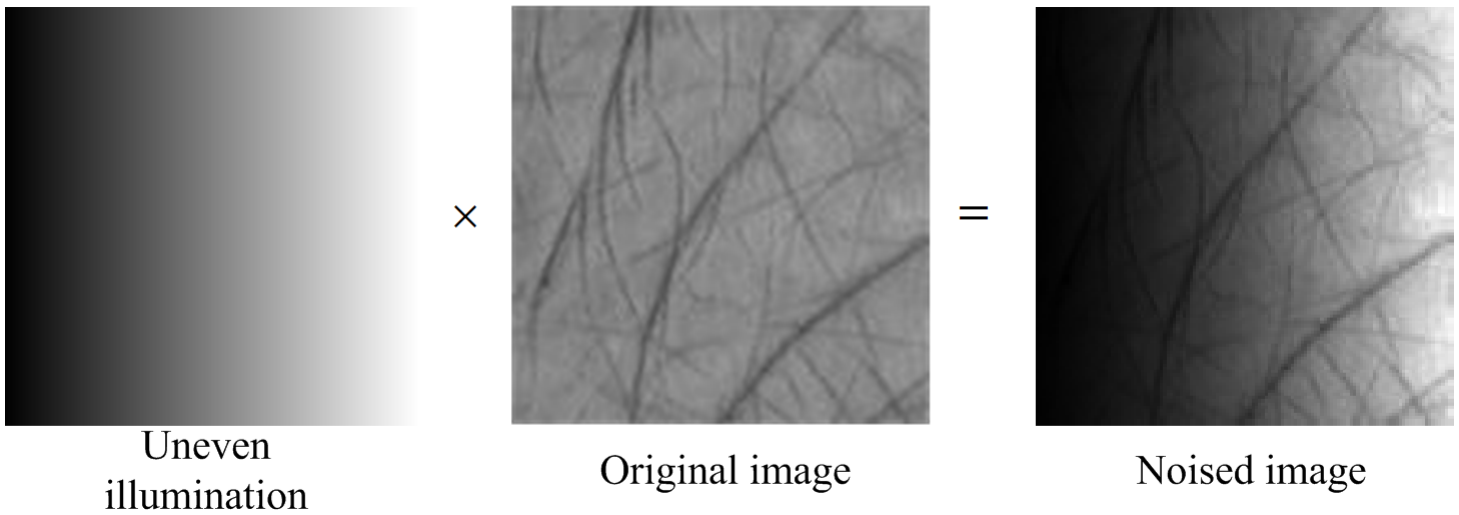
Demonstration of how to generate a noised palmprint image.

All the original images in the database were illuminated uniformly. To verify the robustness of our method against the variation of illumination, we manually generated the noised data through multiplying the palmprint images by an uneven illumination image as shown in Fig 11. In the experiments, the 12000 original palmprint images captured from four spectral bands in the first session were used as the training samples, while the remaining ones with light noise added were taken as the testing samples.

### 4.2 Parameter discussion

**Fig 12:**
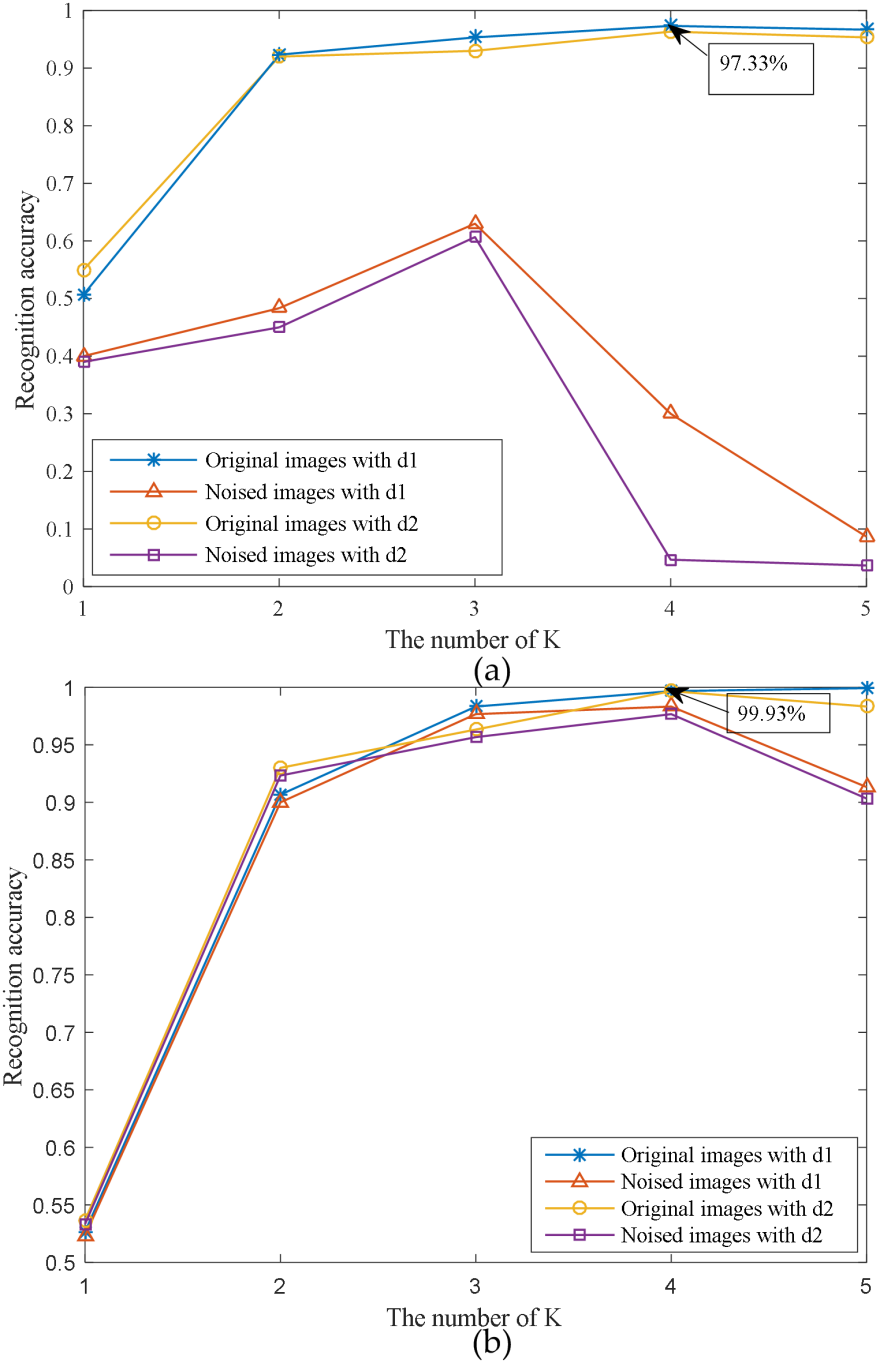
Demonstration of the effects of different settings in FABEMD: (a) experiments without illumination compensation, and (b) experiments with illumination compensation.

We conducted several experiments to investigate the effects of different settings in FABEMD. The results are shown in Fig 12. To test the influence of the number *K* of BIMFs, we gradually increased it from 1 to 5. In the accomplishment of FABEMD, an illumination compensation operation was performed based on the residue. In order to verify its actual performance, a comparison was made between two experiments with and without illumination compensation. We also discussed the results with different ways (*d*_1_ or *d*_2_) of determining the gross window size *w*_*en*−*g*_ for the order-statistics filters. All these trials were carried out on the original images and the noised images, respectively.

From Fig 12(a) and 12(b), we can conclude that the illumination compensation operation significantly improves the robustness of the method against the variation of lighting condition. As shown in Fig 12(a), without this operation, the recognition accuracy decreases seriously when the images are noised by uneven illumination. Meanwhile, when illumination compensation is completed, the results with original images and noised images are nearly the same (Fig 12(b)). It can also be seen that the recognition accuracy increases rapidly as *K* becomes larger. When *K* is 4, the results tend to be optimal. In addition, it is observed that the accuracy by using *d*_1_ as the window size is slightly higher than that by using *d*_2_.

Table 1 lists the recognition accuracies with different parameters in the weighted Fisher criterion. Here, *k* is the sample number in the nearest neighbors for calculating the contribution factor and δ indicates the spread of Gaussian. It can be inferred that the value of *k* has a great influence on the recognition accuracy. As shown in Table 1, in the given range, a larger *k* usually yields a higher recognition accuracy. When *k* = 6 and δ = 5, it produces the highest recognition accuracy.

**Table 1:** Recognition results with different parameters in the weighted Fisher criterion.

	*RA* (%)				
	δ = 4	δ = 5	δ = 6	δ = 7	δ = 8
*k* = 3	68.00	68.67	70.03	67.77	70.53
*k* = 4	87.33	82.97	85.00	86.64	85.33
*k* = 5	93.67	92.34	91.80	93.00	91.67
*k* = 6	98.87	99.50	98.53	97.23	97.23

**Fig 13:**
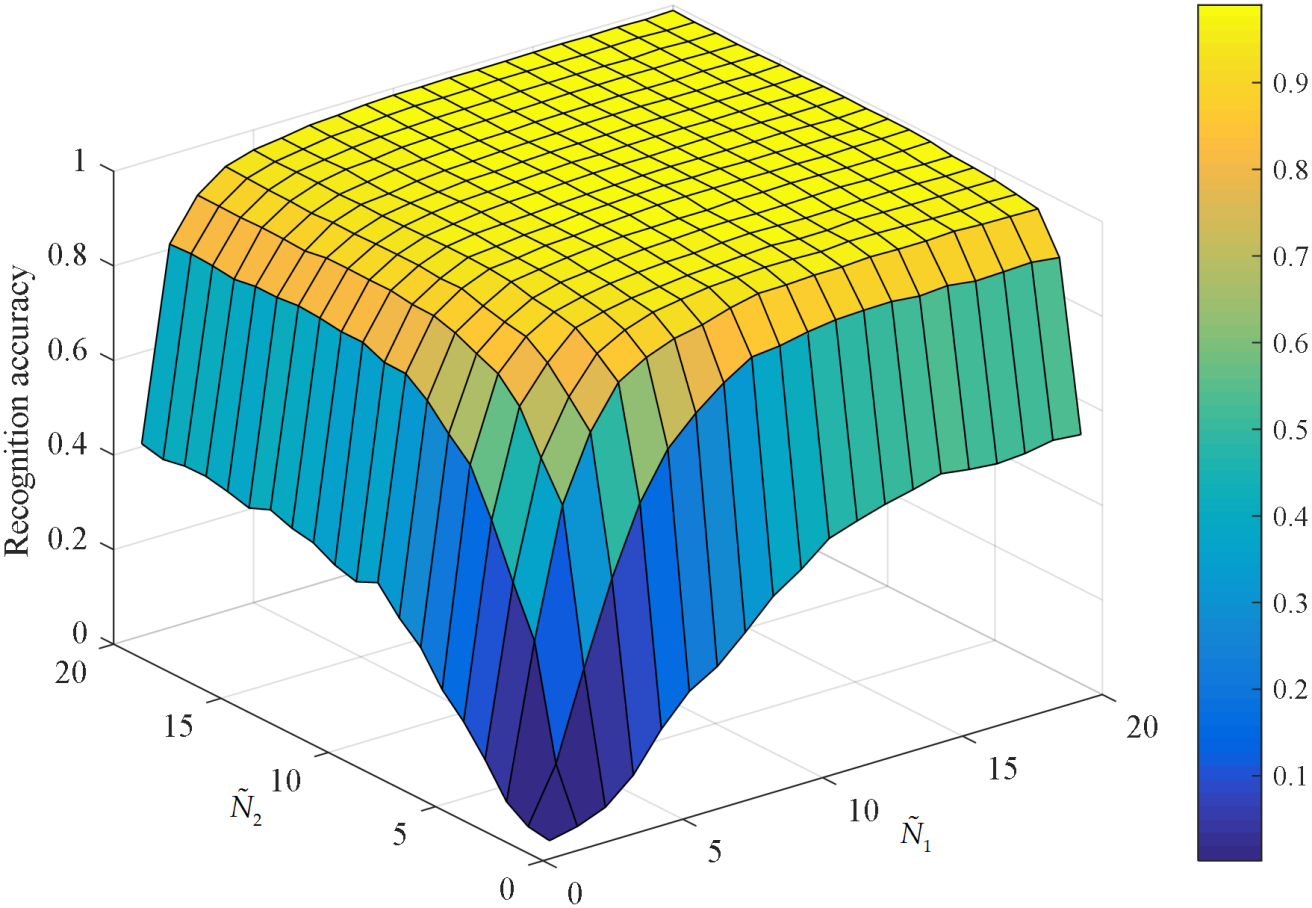
Performance of tensor-based extreme learning machine with different number of hidden nodes.

Fig 13 shows the performance of the tensor-based extreme learning machine with different number of hidden nodes. It is obvious that the recognition accuracy has a growing trend as the number of hidden nodes increases progressively. It converges to the optimal accuracy when the values of ${\tilde{N}}_{1}$ and ${\tilde{N}}_{2}$ are large enough. In our experiments, we set ${\tilde{N}}_{1}=18$ and ${\tilde{N}}_{2}=15$, respectively.

### 4.3 Results analysis of the proposed method

Each spectral band may capture some specific and complementary palm features, providing different information for palmprint recognition. Table 2 illustrates the quantitative results of the proposed method tested with different combinations of the four spectral bands. Some findings can be obtained from the table. In terms of palmprint recognition based on a single spectral band, the Red and NIR bands achieve higher recognition accuracies than the Blue and Green bands. This is because the images captured at Red and NIR spectral bands contain some additional palm vein information, which plays an important role in classifying the images sharing similar palm lines. In addition, it can be observed that the recognition accuracy of fusing multiple spectral bands is higher than that of any single band. While for multispectral fusion, the more bands in fusion do not always achieve a better recognition accuracy. For example, the accuracy of the combination between Red and NIR is 99.47%, which is higher than the result of fusing Blue, Green and NIR bands. We can also find that the performance of the proposed method is seldom affected by the uneven lighting condition.

**Table 2:** Recognition results by different combinations of the spectral bands.

Fused spectral bands	*RA* (%)	
	Original Data	Noised Data
Blue	96.73	96.33
Green	96.93	96.33
Red	97.80	97.37
NIR	97.67	97.03
Blue, Green	99.30	99.13
Blue, Red	99.30	99.23
Blue, NIR	99.10	99.00
Green, Red	99.43	99.30
Green, NIR	99.47	99.13
Red, NIR	99.47	99.50
Blue, Green, Red	99.67	99.47
Blue, Green, NIR	99.27	99.00
Blue, Red, NIR	99.47	99.27
Green, Red, NIR	99.57	99.50
Blue, Green, Red, NIR	99.93	99.50

In order to verify the effectiveness of the proposed fusion strategy, a comparison was made with the sum rule based and the Fisher criterion based image fusion. The results are reported in Table 3. It is evident that for any fusion combination, the proposed weighted Fisher criterion consistently and significantly outperforms both the two comparison methods.

**Table 3:** Performance comparison with different fusion rules.

Fused spectral bands	*RA* (%)		
	Sum Rule	Fisher Criterion	Weighted Fisher Criterion
Blue, Green	93.27	97.87	99.13
Blue, Red	93.47	98.40	99.23
Blue, NIR	93.27	97.80	99.00
Green, Red	93.77	98.47	99.30
Green, NIR	93.60	97.93	99.13
Red, NIR	93.43	98.60	99.50
Blue, Green, Red	93.17	98.43	99.47
Blue, Green, NIR	93.30	98.10	99.00
Blue, Red, NIR	93.37	98.13	99.27
Green, Red, NIR	93.77	98.47	99.50
Blue, Green, Red, NIR	93.87	98.63	99.50

Another comparison was made by using different classifiers. The KNN, ELM and TELM were compared in terms of recognition accuracy and computational time. Table 4 depicts the recognition accuracies with different fusion combinations. It can be found that the TELM yields the highest recognition accuracy. For any spectral band combination, the result of TELM is much higher than that of ELM. So we can conclude the TELM is an effective improvement of ELM. Compared with KNN, the TELM also maintains an obvious advantage. As for the computational cost shown in Table 5 (Here, the time is referred to as the computational time for the entire database), it can be seen that the TELM costs the least computational time. In comparison with ELM, the TELM tends to be optimized with fewer hidden nodes, resulting in much less computational time. Although the KNN does not need a training process, it executes a matching operation with each reference sample when classification, making it the most complicated to be calculated. Overall, the TELM outperforms both the KNN and ELM.

**Table 4:** Performance comparison with different classifiers.

Fused spectral bands	*RA* (%)		
	KNN	ELM	TELM
Blue, Green	97.10	78.53	99.13
Blue, Red	97.37	75.03	99.23
Blue, NIR	95.70	75.73	99.00
Green, Red	98.57	73.67	99.30
Green, NIR	98.03	72.40	99.13
Red, NIR	98.63	72.37	99.50
Blue, Green, Red	97.97	75.40	99.47
Blue, Green, NIR	96.75	76.87	99.00
Blue, Red, NIR	97.63	74.27	99.27
Green, Red, NIR	98.70	75.43	99.50
Blue, Green, Red, NIR	98.03	75.77	99.50

**Table 5:** Computational time of different classifiers.

Methods	Training Time (s)	Testing Time (s)	Total Time (s)
KNN	-	118.88	118.88
ELM	51.41	13.95	65.36
TELM	14.43	2.54	16.97

**Fig 14:**
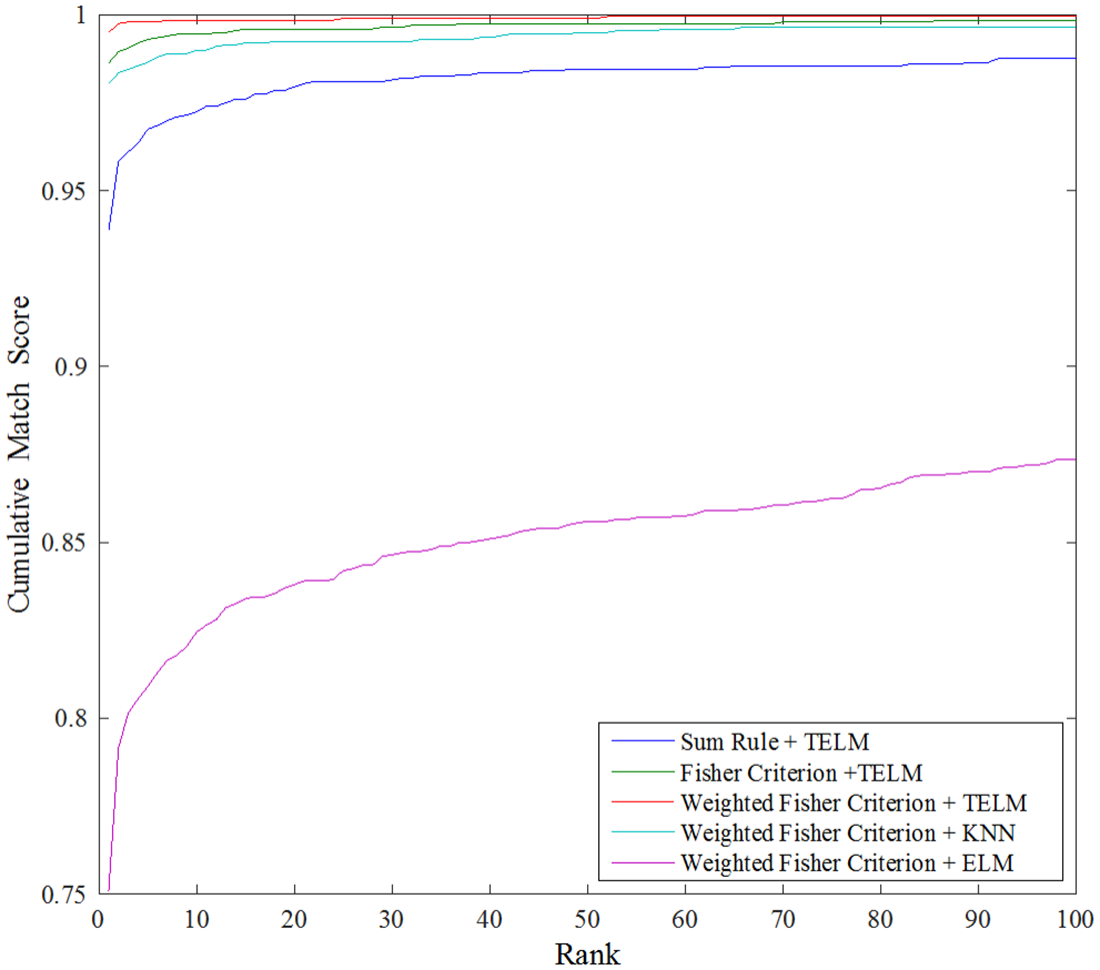
Performance comparison of different fusion and classification methods in terms of Cumulative Match Characteristic curves.

In order to further evaluate the proposed fusion rule and classification method, some Cumulative Match Characteristic curves were generated by using the sum rule, the Fisher criterion, the weighted Fisher criterion for image fusion and the TELM, the KNN, the ELM for classification, respectively. Seen from Fig 14, we can find that the proposed method (weighted Fisher criterion + TELM) has the highest rank-1 recognition accuracy. At the same time, it is more towards the upper left corner of the plots compared with the other methods. So we can conclude that the weighted Fisher criterion based image fusion and the TELM classifier are superior to all the other methods, which is quite consistent with the results reported in Tables 3 and 4.

Table 6 shows the comparison results with some state-of-art multispectral palmprint recognition methods, including an image-level fusion method, a matching score-level fusion method and a quaternion matrix based method. We can find that the performance of the method in [25] degrades seriously when the palmprint images are noised with uneven illumination. Although the methods in [11] and [21] are rarely affected by the illumination change, they can’t provide a recognition accuracy as high as ours. The proposed method can attain the highest recognition accuracy under even or uneven illumination conditions among the four methods.

**Table 6:** Performance comparison with different multispectral palmprint recognition methods.

Methods	*RA* (%)	
	Original Data	Noised Data
Image-level fusion by DWT [11]	99.03	98.53
Matching score-level fusion by line orientation code [21]	99.43	98.70
QPCA+QDWT [25]	98.83	65.63
Our proposed method	99.93	99.50

Table 7 gives the average execution time for each step when using the proposed method to recognize the identity of a single multispectral palmprint sample. It should be noted that the results for image fusion and palmprint classification are the testing time with the fusion coefficients and the TELM model calculated in advance. Actually, these parameters only need to be computed once and the corresponding computational times are meaningless. As shown in the table, the proposed method is fast enough for real-time applications.

**Table 7:** Time cost of the proposed method.

	Average time (ms)
FABEMD	187*4
Image fusion	1.1
Palmprint classification	0.8
Total time	749.9

## 5 Conclusions

In this paper, we have investigated an illumination-invariant multispectral palmprint recognition method. It combined the information across multiple spectral bands (Blue, Green, Red and NIR) by performing a fusion at image level. Each image captured at a single spectral band was decomposed into several BIMFs and a residue using FABEMD. Then the residue was used to estimate the illumination condition of the palmprint, based on which the BIMFs were adjusted. To guarantee the final recognition accuracy of images in the fusion space as high as possible, a weighted Fisher criterion considering the different contributions of image samples was proposed to find the fusion coefficients. Furthermore, an improved extreme learning machine based on tensor decomposition was utilized for feature extraction and classification. It occupied a higher-order singular value decomposition technique to determine the input weights of a single-hidden-layer feedforward neural network, which could fully maintain the structure features of two-dimensional signals. Experiments carried out on the PolyU multispectral palmprint database under different illumination conditions showed that our proposed method could achieve very competitive results with great robustness against illumination variation.

## Supporting information

S1 File All relevant data. (ZIP)
