# Supplementary figures and images for: Multispectral image fusion for illumination-invariant palmprint recognition (part 2 of 2)
Source: PLoS One. 2017 May 30;12(5):e0178432. doi: 10.1371/journal.pone.0178432 (PMC5448787; doi:10.1371/journal.pone.0178432)

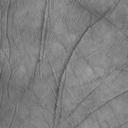

Supplement: S1 File — (ZIP) [file pone.0178432.s001.zip › Palmprint/B_0009_05.jpg]

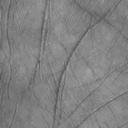

Supplement: S1 File — (ZIP) [file pone.0178432.s001.zip › Palmprint/B_0009_06.jpg]

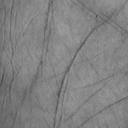

Supplement: S1 File — (ZIP) [file pone.0178432.s001.zip › Palmprint/B_0009_07.jpg]

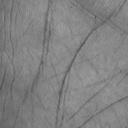

Supplement: S1 File — (ZIP) [file pone.0178432.s001.zip › Palmprint/B_0009_08.jpg]

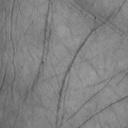

Supplement: S1 File — (ZIP) [file pone.0178432.s001.zip › Palmprint/B_0009_09.jpg]

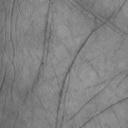

Supplement: S1 File — (ZIP) [file pone.0178432.s001.zip › Palmprint/B_0009_10.jpg]

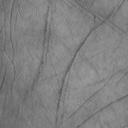

Supplement: S1 File — (ZIP) [file pone.0178432.s001.zip › Palmprint/B_0009_11.jpg]

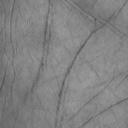

Supplement: S1 File — (ZIP) [file pone.0178432.s001.zip › Palmprint/B_0009_12.jpg]

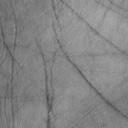

Supplement: S1 File — (ZIP) [file pone.0178432.s001.zip › Palmprint/B_0010_01.jpg]

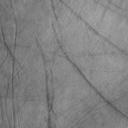

Supplement: S1 File — (ZIP) [file pone.0178432.s001.zip › Palmprint/B_0010_02.jpg]

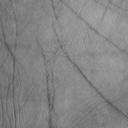

Supplement: S1 File — (ZIP) [file pone.0178432.s001.zip › Palmprint/B_0010_03.jpg]

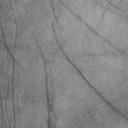

Supplement: S1 File — (ZIP) [file pone.0178432.s001.zip › Palmprint/B_0010_04.jpg]

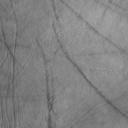

Supplement: S1 File — (ZIP) [file pone.0178432.s001.zip › Palmprint/B_0010_05.jpg]

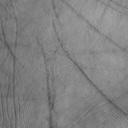

Supplement: S1 File — (ZIP) [file pone.0178432.s001.zip › Palmprint/B_0010_06.jpg]

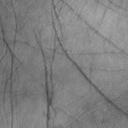

Supplement: S1 File — (ZIP) [file pone.0178432.s001.zip › Palmprint/B_0010_07.jpg]

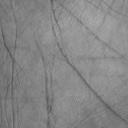

Supplement: S1 File — (ZIP) [file pone.0178432.s001.zip › Palmprint/B_0010_08.jpg]

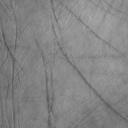

Supplement: S1 File — (ZIP) [file pone.0178432.s001.zip › Palmprint/B_0010_09.jpg]

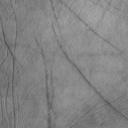

Supplement: S1 File — (ZIP) [file pone.0178432.s001.zip › Palmprint/B_0010_10.jpg]

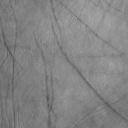

Supplement: S1 File — (ZIP) [file pone.0178432.s001.zip › Palmprint/B_0010_11.jpg]

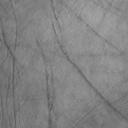

Supplement: S1 File — (ZIP) [file pone.0178432.s001.zip › Palmprint/B_0010_12.jpg]

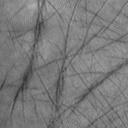

Supplement: S1 File — (ZIP) [file pone.0178432.s001.zip › Palmprint/B_0011_01.jpg]

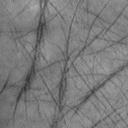

Supplement: S1 File — (ZIP) [file pone.0178432.s001.zip › Palmprint/B_0011_02.jpg]

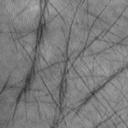

Supplement: S1 File — (ZIP) [file pone.0178432.s001.zip › Palmprint/B_0011_03.jpg]

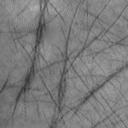

Supplement: S1 File — (ZIP) [file pone.0178432.s001.zip › Palmprint/B_0011_04.jpg]

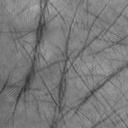

Supplement: S1 File — (ZIP) [file pone.0178432.s001.zip › Palmprint/B_0011_05.jpg]

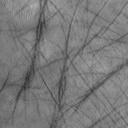

Supplement: S1 File — (ZIP) [file pone.0178432.s001.zip › Palmprint/B_0011_06.jpg]

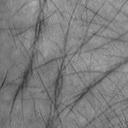

Supplement: S1 File — (ZIP) [file pone.0178432.s001.zip › Palmprint/B_0011_07.jpg]

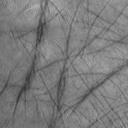

Supplement: S1 File — (ZIP) [file pone.0178432.s001.zip › Palmprint/B_0011_08.jpg]

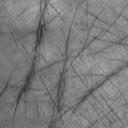

Supplement: S1 File — (ZIP) [file pone.0178432.s001.zip › Palmprint/B_0011_09.jpg]

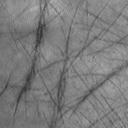

Supplement: S1 File — (ZIP) [file pone.0178432.s001.zip › Palmprint/B_0011_10.jpg]

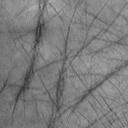

Supplement: S1 File — (ZIP) [file pone.0178432.s001.zip › Palmprint/B_0011_11.jpg]

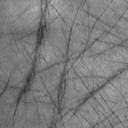

Supplement: S1 File — (ZIP) [file pone.0178432.s001.zip › Palmprint/B_0011_12.jpg]

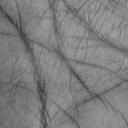

Supplement: S1 File — (ZIP) [file pone.0178432.s001.zip › Palmprint/B_0012_01.jpg]

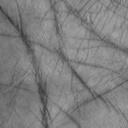

Supplement: S1 File — (ZIP) [file pone.0178432.s001.zip › Palmprint/B_0012_02.jpg]

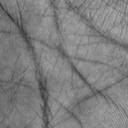

Supplement: S1 File — (ZIP) [file pone.0178432.s001.zip › Palmprint/B_0012_03.jpg]

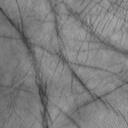

Supplement: S1 File — (ZIP) [file pone.0178432.s001.zip › Palmprint/B_0012_04.jpg]

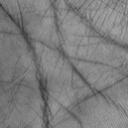

Supplement: S1 File — (ZIP) [file pone.0178432.s001.zip › Palmprint/B_0012_05.jpg]

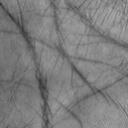

Supplement: S1 File — (ZIP) [file pone.0178432.s001.zip › Palmprint/B_0012_06.jpg]

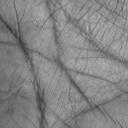

Supplement: S1 File — (ZIP) [file pone.0178432.s001.zip › Palmprint/B_0012_07.jpg]

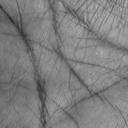

Supplement: S1 File — (ZIP) [file pone.0178432.s001.zip › Palmprint/B_0012_08.jpg]

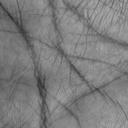

Supplement: S1 File — (ZIP) [file pone.0178432.s001.zip › Palmprint/B_0012_09.jpg]

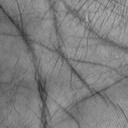

Supplement: S1 File — (ZIP) [file pone.0178432.s001.zip › Palmprint/B_0012_10.jpg]

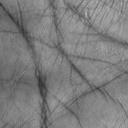

Supplement: S1 File — (ZIP) [file pone.0178432.s001.zip › Palmprint/B_0012_11.jpg]

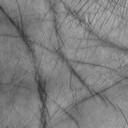

Supplement: S1 File — (ZIP) [file pone.0178432.s001.zip › Palmprint/B_0012_12.jpg]

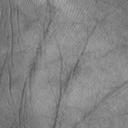

Supplement: S1 File — (ZIP) [file pone.0178432.s001.zip › Palmprint/B_0013_01.jpg]

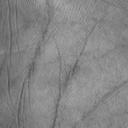

Supplement: S1 File — (ZIP) [file pone.0178432.s001.zip › Palmprint/B_0013_02.jpg]

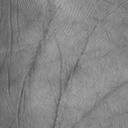

Supplement: S1 File — (ZIP) [file pone.0178432.s001.zip › Palmprint/B_0013_03.jpg]

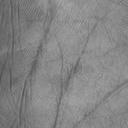

Supplement: S1 File — (ZIP) [file pone.0178432.s001.zip › Palmprint/B_0013_04.jpg]

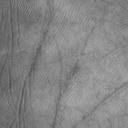

Supplement: S1 File — (ZIP) [file pone.0178432.s001.zip › Palmprint/B_0013_05.jpg]

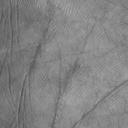

Supplement: S1 File — (ZIP) [file pone.0178432.s001.zip › Palmprint/B_0013_06.jpg]

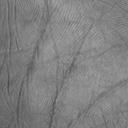

Supplement: S1 File — (ZIP) [file pone.0178432.s001.zip › Palmprint/B_0013_07.jpg]

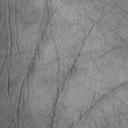

Supplement: S1 File — (ZIP) [file pone.0178432.s001.zip › Palmprint/B_0013_08.jpg]

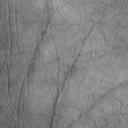

Supplement: S1 File — (ZIP) [file pone.0178432.s001.zip › Palmprint/B_0013_09.jpg]

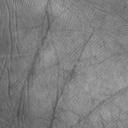

Supplement: S1 File — (ZIP) [file pone.0178432.s001.zip › Palmprint/B_0013_10.jpg]

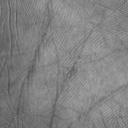

Supplement: S1 File — (ZIP) [file pone.0178432.s001.zip › Palmprint/B_0013_11.jpg]

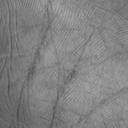

Supplement: S1 File — (ZIP) [file pone.0178432.s001.zip › Palmprint/B_0013_12.jpg]

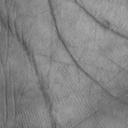

Supplement: S1 File — (ZIP) [file pone.0178432.s001.zip › Palmprint/B_0014_01.jpg]

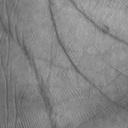

Supplement: S1 File — (ZIP) [file pone.0178432.s001.zip › Palmprint/B_0014_02.jpg]

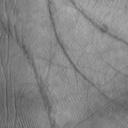

Supplement: S1 File — (ZIP) [file pone.0178432.s001.zip › Palmprint/B_0014_03.jpg]

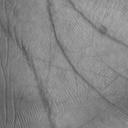

Supplement: S1 File — (ZIP) [file pone.0178432.s001.zip › Palmprint/B_0014_04.jpg]

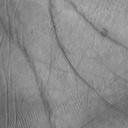

Supplement: S1 File — (ZIP) [file pone.0178432.s001.zip › Palmprint/B_0014_05.jpg]

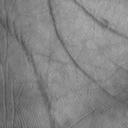

Supplement: S1 File — (ZIP) [file pone.0178432.s001.zip › Palmprint/B_0014_06.jpg]

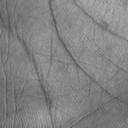

Supplement: S1 File — (ZIP) [file pone.0178432.s001.zip › Palmprint/B_0014_07.jpg]

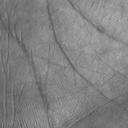

Supplement: S1 File — (ZIP) [file pone.0178432.s001.zip › Palmprint/B_0014_08.jpg]

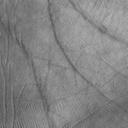

Supplement: S1 File — (ZIP) [file pone.0178432.s001.zip › Palmprint/B_0014_09.jpg]

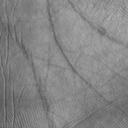

Supplement: S1 File — (ZIP) [file pone.0178432.s001.zip › Palmprint/B_0014_10.jpg]

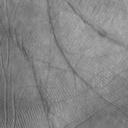

Supplement: S1 File — (ZIP) [file pone.0178432.s001.zip › Palmprint/B_0014_11.jpg]

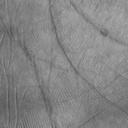

Supplement: S1 File — (ZIP) [file pone.0178432.s001.zip › Palmprint/B_0014_12.jpg]

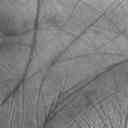

Supplement: S1 File — (ZIP) [file pone.0178432.s001.zip › Palmprint/B_0015_01.jpg]

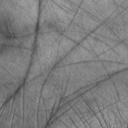

Supplement: S1 File — (ZIP) [file pone.0178432.s001.zip › Palmprint/B_0015_02.jpg]

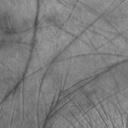

Supplement: S1 File — (ZIP) [file pone.0178432.s001.zip › Palmprint/B_0015_03.jpg]

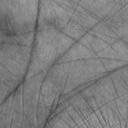

Supplement: S1 File — (ZIP) [file pone.0178432.s001.zip › Palmprint/B_0015_04.jpg]

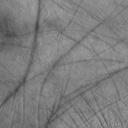

Supplement: S1 File — (ZIP) [file pone.0178432.s001.zip › Palmprint/B_0015_05.jpg]

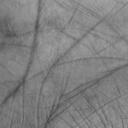

Supplement: S1 File — (ZIP) [file pone.0178432.s001.zip › Palmprint/B_0015_06.jpg]

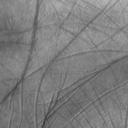

Supplement: S1 File — (ZIP) [file pone.0178432.s001.zip › Palmprint/B_0015_07.jpg]

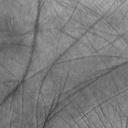

Supplement: S1 File — (ZIP) [file pone.0178432.s001.zip › Palmprint/B_0015_08.jpg]

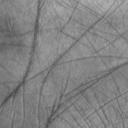

Supplement: S1 File — (ZIP) [file pone.0178432.s001.zip › Palmprint/B_0015_09.jpg]

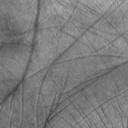

Supplement: S1 File — (ZIP) [file pone.0178432.s001.zip › Palmprint/B_0015_10.jpg]

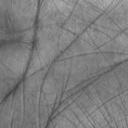

Supplement: S1 File — (ZIP) [file pone.0178432.s001.zip › Palmprint/B_0015_11.jpg]

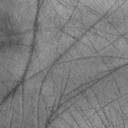

Supplement: S1 File — (ZIP) [file pone.0178432.s001.zip › Palmprint/B_0015_12.jpg]

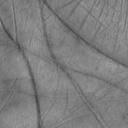

Supplement: S1 File — (ZIP) [file pone.0178432.s001.zip › Palmprint/B_0016_01.jpg]

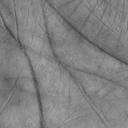

Supplement: S1 File — (ZIP) [file pone.0178432.s001.zip › Palmprint/B_0016_02.jpg]

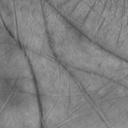

Supplement: S1 File — (ZIP) [file pone.0178432.s001.zip › Palmprint/B_0016_03.jpg]

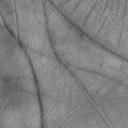

Supplement: S1 File — (ZIP) [file pone.0178432.s001.zip › Palmprint/B_0016_04.jpg]

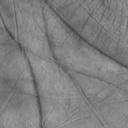

Supplement: S1 File — (ZIP) [file pone.0178432.s001.zip › Palmprint/B_0016_05.jpg]

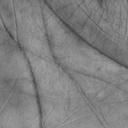

Supplement: S1 File — (ZIP) [file pone.0178432.s001.zip › Palmprint/B_0016_06.jpg]

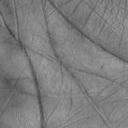

Supplement: S1 File — (ZIP) [file pone.0178432.s001.zip › Palmprint/B_0016_07.jpg]

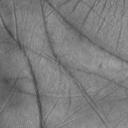

Supplement: S1 File — (ZIP) [file pone.0178432.s001.zip › Palmprint/B_0016_08.jpg]

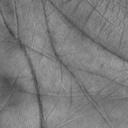

Supplement: S1 File — (ZIP) [file pone.0178432.s001.zip › Palmprint/B_0016_09.jpg]

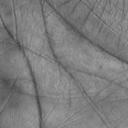

Supplement: S1 File — (ZIP) [file pone.0178432.s001.zip › Palmprint/B_0016_10.jpg]

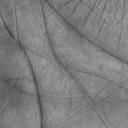

Supplement: S1 File — (ZIP) [file pone.0178432.s001.zip › Palmprint/B_0016_11.jpg]

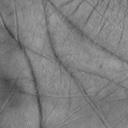

Supplement: S1 File — (ZIP) [file pone.0178432.s001.zip › Palmprint/B_0016_12.jpg]

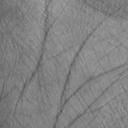

Supplement: S1 File — (ZIP) [file pone.0178432.s001.zip › Palmprint/B_0017_01.jpg]

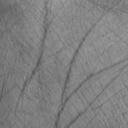

Supplement: S1 File — (ZIP) [file pone.0178432.s001.zip › Palmprint/B_0017_02.jpg]

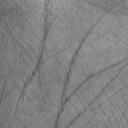

Supplement: S1 File — (ZIP) [file pone.0178432.s001.zip › Palmprint/B_0017_03.jpg]

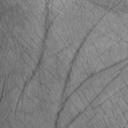

Supplement: S1 File — (ZIP) [file pone.0178432.s001.zip › Palmprint/B_0017_04.jpg]

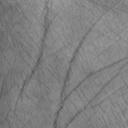

Supplement: S1 File — (ZIP) [file pone.0178432.s001.zip › Palmprint/B_0017_05.jpg]

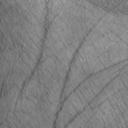

Supplement: S1 File — (ZIP) [file pone.0178432.s001.zip › Palmprint/B_0017_06.jpg]

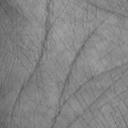

Supplement: S1 File — (ZIP) [file pone.0178432.s001.zip › Palmprint/B_0017_07.jpg]

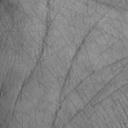

Supplement: S1 File — (ZIP) [file pone.0178432.s001.zip › Palmprint/B_0017_08.jpg]
