# Supplementary figures and images for: Multispectral image fusion for illumination-invariant palmprint recognition (part 1 of 2)
Source: PLoS One. 2017 May 30;12(5):e0178432. doi: 10.1371/journal.pone.0178432 (PMC5448787; doi:10.1371/journal.pone.0178432)

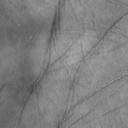

Supplement: S1 File — (ZIP) [file pone.0178432.s001.zip › Palmprint/B_0001_01.jpg]

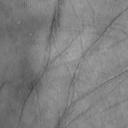

Supplement: S1 File — (ZIP) [file pone.0178432.s001.zip › Palmprint/B_0001_02.jpg]

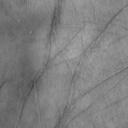

Supplement: S1 File — (ZIP) [file pone.0178432.s001.zip › Palmprint/B_0001_03.jpg]

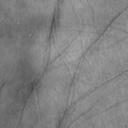

Supplement: S1 File — (ZIP) [file pone.0178432.s001.zip › Palmprint/B_0001_04.jpg]

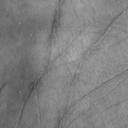

Supplement: S1 File — (ZIP) [file pone.0178432.s001.zip › Palmprint/B_0001_05.jpg]

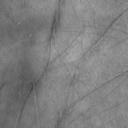

Supplement: S1 File — (ZIP) [file pone.0178432.s001.zip › Palmprint/B_0001_06.jpg]

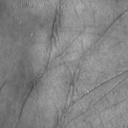

Supplement: S1 File — (ZIP) [file pone.0178432.s001.zip › Palmprint/B_0001_07.jpg]

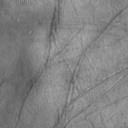

Supplement: S1 File — (ZIP) [file pone.0178432.s001.zip › Palmprint/B_0001_08.jpg]

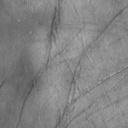

Supplement: S1 File — (ZIP) [file pone.0178432.s001.zip › Palmprint/B_0001_09.jpg]

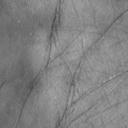

Supplement: S1 File — (ZIP) [file pone.0178432.s001.zip › Palmprint/B_0001_10.jpg]

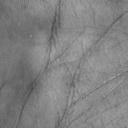

Supplement: S1 File — (ZIP) [file pone.0178432.s001.zip › Palmprint/B_0001_11.jpg]

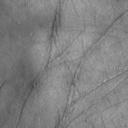

Supplement: S1 File — (ZIP) [file pone.0178432.s001.zip › Palmprint/B_0001_12.jpg]

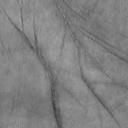

Supplement: S1 File — (ZIP) [file pone.0178432.s001.zip › Palmprint/B_0002_01.jpg]

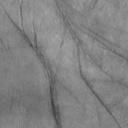

Supplement: S1 File — (ZIP) [file pone.0178432.s001.zip › Palmprint/B_0002_02.jpg]

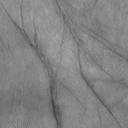

Supplement: S1 File — (ZIP) [file pone.0178432.s001.zip › Palmprint/B_0002_03.jpg]

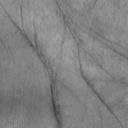

Supplement: S1 File — (ZIP) [file pone.0178432.s001.zip › Palmprint/B_0002_04.jpg]

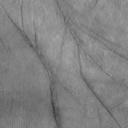

Supplement: S1 File — (ZIP) [file pone.0178432.s001.zip › Palmprint/B_0002_05.jpg]

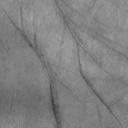

Supplement: S1 File — (ZIP) [file pone.0178432.s001.zip › Palmprint/B_0002_06.jpg]

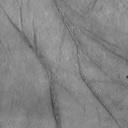

Supplement: S1 File — (ZIP) [file pone.0178432.s001.zip › Palmprint/B_0002_07.jpg]

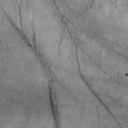

Supplement: S1 File — (ZIP) [file pone.0178432.s001.zip › Palmprint/B_0002_08.jpg]

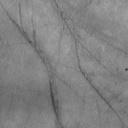

Supplement: S1 File — (ZIP) [file pone.0178432.s001.zip › Palmprint/B_0002_09.jpg]

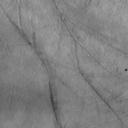

Supplement: S1 File — (ZIP) [file pone.0178432.s001.zip › Palmprint/B_0002_10.jpg]

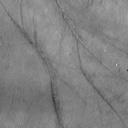

Supplement: S1 File — (ZIP) [file pone.0178432.s001.zip › Palmprint/B_0002_11.jpg]

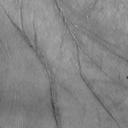

Supplement: S1 File — (ZIP) [file pone.0178432.s001.zip › Palmprint/B_0002_12.jpg]

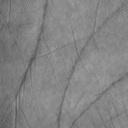

Supplement: S1 File — (ZIP) [file pone.0178432.s001.zip › Palmprint/B_0003_01.jpg]

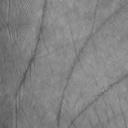

Supplement: S1 File — (ZIP) [file pone.0178432.s001.zip › Palmprint/B_0003_02.jpg]

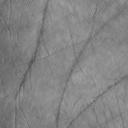

Supplement: S1 File — (ZIP) [file pone.0178432.s001.zip › Palmprint/B_0003_03.jpg]

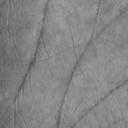

Supplement: S1 File — (ZIP) [file pone.0178432.s001.zip › Palmprint/B_0003_04.jpg]

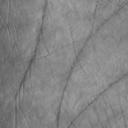

Supplement: S1 File — (ZIP) [file pone.0178432.s001.zip › Palmprint/B_0003_05.jpg]

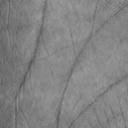

Supplement: S1 File — (ZIP) [file pone.0178432.s001.zip › Palmprint/B_0003_06.jpg]

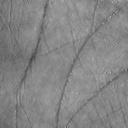

Supplement: S1 File — (ZIP) [file pone.0178432.s001.zip › Palmprint/B_0003_07.jpg]

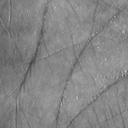

Supplement: S1 File — (ZIP) [file pone.0178432.s001.zip › Palmprint/B_0003_08.jpg]

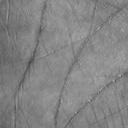

Supplement: S1 File — (ZIP) [file pone.0178432.s001.zip › Palmprint/B_0003_09.jpg]

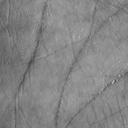

Supplement: S1 File — (ZIP) [file pone.0178432.s001.zip › Palmprint/B_0003_10.jpg]

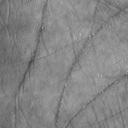

Supplement: S1 File — (ZIP) [file pone.0178432.s001.zip › Palmprint/B_0003_11.jpg]

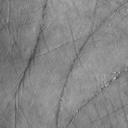

Supplement: S1 File — (ZIP) [file pone.0178432.s001.zip › Palmprint/B_0003_12.jpg]

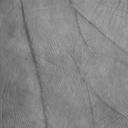

Supplement: S1 File — (ZIP) [file pone.0178432.s001.zip › Palmprint/B_0004_01.jpg]

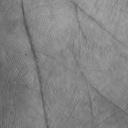

Supplement: S1 File — (ZIP) [file pone.0178432.s001.zip › Palmprint/B_0004_02.jpg]

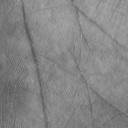

Supplement: S1 File — (ZIP) [file pone.0178432.s001.zip › Palmprint/B_0004_03.jpg]

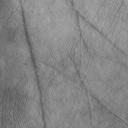

Supplement: S1 File — (ZIP) [file pone.0178432.s001.zip › Palmprint/B_0004_04.jpg]

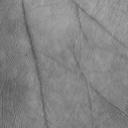

Supplement: S1 File — (ZIP) [file pone.0178432.s001.zip › Palmprint/B_0004_05.jpg]

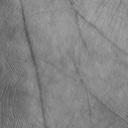

Supplement: S1 File — (ZIP) [file pone.0178432.s001.zip › Palmprint/B_0004_06.jpg]

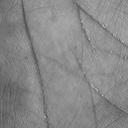

Supplement: S1 File — (ZIP) [file pone.0178432.s001.zip › Palmprint/B_0004_07.jpg]

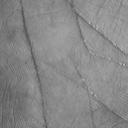

Supplement: S1 File — (ZIP) [file pone.0178432.s001.zip › Palmprint/B_0004_08.jpg]

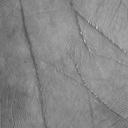

Supplement: S1 File — (ZIP) [file pone.0178432.s001.zip › Palmprint/B_0004_09.jpg]

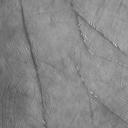

Supplement: S1 File — (ZIP) [file pone.0178432.s001.zip › Palmprint/B_0004_10.jpg]

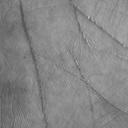

Supplement: S1 File — (ZIP) [file pone.0178432.s001.zip › Palmprint/B_0004_11.jpg]

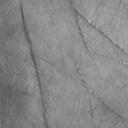

Supplement: S1 File — (ZIP) [file pone.0178432.s001.zip › Palmprint/B_0004_12.jpg]

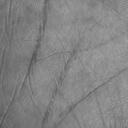

Supplement: S1 File — (ZIP) [file pone.0178432.s001.zip › Palmprint/B_0005_01.jpg]

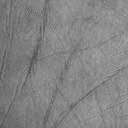

Supplement: S1 File — (ZIP) [file pone.0178432.s001.zip › Palmprint/B_0005_02.jpg]

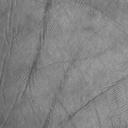

Supplement: S1 File — (ZIP) [file pone.0178432.s001.zip › Palmprint/B_0005_03.jpg]

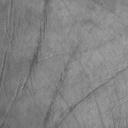

Supplement: S1 File — (ZIP) [file pone.0178432.s001.zip › Palmprint/B_0005_04.jpg]

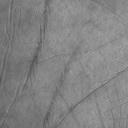

Supplement: S1 File — (ZIP) [file pone.0178432.s001.zip › Palmprint/B_0005_05.jpg]

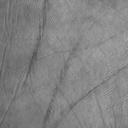

Supplement: S1 File — (ZIP) [file pone.0178432.s001.zip › Palmprint/B_0005_06.jpg]

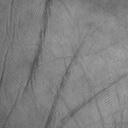

Supplement: S1 File — (ZIP) [file pone.0178432.s001.zip › Palmprint/B_0005_07.jpg]

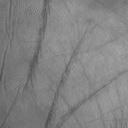

Supplement: S1 File — (ZIP) [file pone.0178432.s001.zip › Palmprint/B_0005_08.jpg]

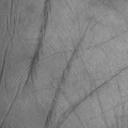

Supplement: S1 File — (ZIP) [file pone.0178432.s001.zip › Palmprint/B_0005_09.jpg]

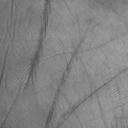

Supplement: S1 File — (ZIP) [file pone.0178432.s001.zip › Palmprint/B_0005_10.jpg]

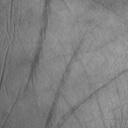

Supplement: S1 File — (ZIP) [file pone.0178432.s001.zip › Palmprint/B_0005_11.jpg]

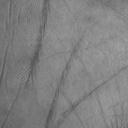

Supplement: S1 File — (ZIP) [file pone.0178432.s001.zip › Palmprint/B_0005_12.jpg]

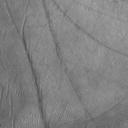

Supplement: S1 File — (ZIP) [file pone.0178432.s001.zip › Palmprint/B_0006_01.jpg]

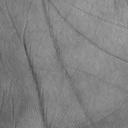

Supplement: S1 File — (ZIP) [file pone.0178432.s001.zip › Palmprint/B_0006_02.jpg]

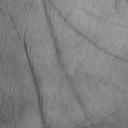

Supplement: S1 File — (ZIP) [file pone.0178432.s001.zip › Palmprint/B_0006_03.jpg]

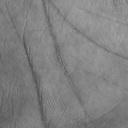

Supplement: S1 File — (ZIP) [file pone.0178432.s001.zip › Palmprint/B_0006_04.jpg]

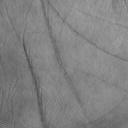

Supplement: S1 File — (ZIP) [file pone.0178432.s001.zip › Palmprint/B_0006_05.jpg]

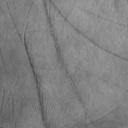

Supplement: S1 File — (ZIP) [file pone.0178432.s001.zip › Palmprint/B_0006_06.jpg]

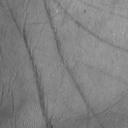

Supplement: S1 File — (ZIP) [file pone.0178432.s001.zip › Palmprint/B_0006_07.jpg]

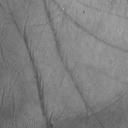

Supplement: S1 File — (ZIP) [file pone.0178432.s001.zip › Palmprint/B_0006_08.jpg]

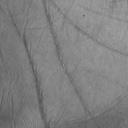

Supplement: S1 File — (ZIP) [file pone.0178432.s001.zip › Palmprint/B_0006_09.jpg]

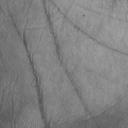

Supplement: S1 File — (ZIP) [file pone.0178432.s001.zip › Palmprint/B_0006_10.jpg]

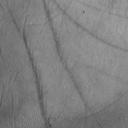

Supplement: S1 File — (ZIP) [file pone.0178432.s001.zip › Palmprint/B_0006_11.jpg]

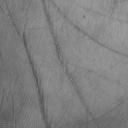

Supplement: S1 File — (ZIP) [file pone.0178432.s001.zip › Palmprint/B_0006_12.jpg]

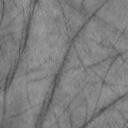

Supplement: S1 File — (ZIP) [file pone.0178432.s001.zip › Palmprint/B_0007_01.jpg]

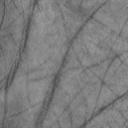

Supplement: S1 File — (ZIP) [file pone.0178432.s001.zip › Palmprint/B_0007_02.jpg]

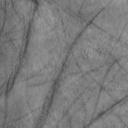

Supplement: S1 File — (ZIP) [file pone.0178432.s001.zip › Palmprint/B_0007_03.jpg]

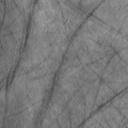

Supplement: S1 File — (ZIP) [file pone.0178432.s001.zip › Palmprint/B_0007_04.jpg]

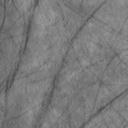

Supplement: S1 File — (ZIP) [file pone.0178432.s001.zip › Palmprint/B_0007_05.jpg]

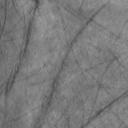

Supplement: S1 File — (ZIP) [file pone.0178432.s001.zip › Palmprint/B_0007_06.jpg]

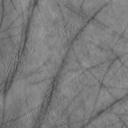

Supplement: S1 File — (ZIP) [file pone.0178432.s001.zip › Palmprint/B_0007_07.jpg]

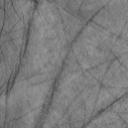

Supplement: S1 File — (ZIP) [file pone.0178432.s001.zip › Palmprint/B_0007_08.jpg]

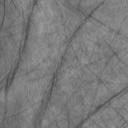

Supplement: S1 File — (ZIP) [file pone.0178432.s001.zip › Palmprint/B_0007_09.jpg]

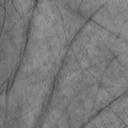

Supplement: S1 File — (ZIP) [file pone.0178432.s001.zip › Palmprint/B_0007_10.jpg]

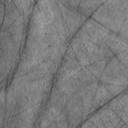

Supplement: S1 File — (ZIP) [file pone.0178432.s001.zip › Palmprint/B_0007_11.jpg]

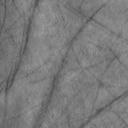

Supplement: S1 File — (ZIP) [file pone.0178432.s001.zip › Palmprint/B_0007_12.jpg]

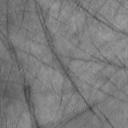

Supplement: S1 File — (ZIP) [file pone.0178432.s001.zip › Palmprint/B_0008_01.jpg]

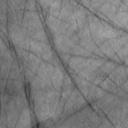

Supplement: S1 File — (ZIP) [file pone.0178432.s001.zip › Palmprint/B_0008_02.jpg]

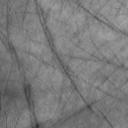

Supplement: S1 File — (ZIP) [file pone.0178432.s001.zip › Palmprint/B_0008_03.jpg]

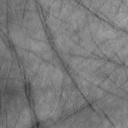

Supplement: S1 File — (ZIP) [file pone.0178432.s001.zip › Palmprint/B_0008_04.jpg]

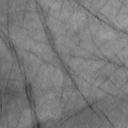

Supplement: S1 File — (ZIP) [file pone.0178432.s001.zip › Palmprint/B_0008_05.jpg]

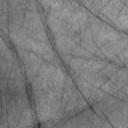

Supplement: S1 File — (ZIP) [file pone.0178432.s001.zip › Palmprint/B_0008_06.jpg]

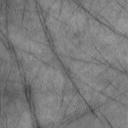

Supplement: S1 File — (ZIP) [file pone.0178432.s001.zip › Palmprint/B_0008_07.jpg]

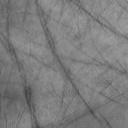

Supplement: S1 File — (ZIP) [file pone.0178432.s001.zip › Palmprint/B_0008_08.jpg]

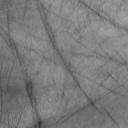

Supplement: S1 File — (ZIP) [file pone.0178432.s001.zip › Palmprint/B_0008_09.jpg]

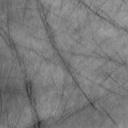

Supplement: S1 File — (ZIP) [file pone.0178432.s001.zip › Palmprint/B_0008_10.jpg]

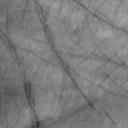

Supplement: S1 File — (ZIP) [file pone.0178432.s001.zip › Palmprint/B_0008_11.jpg]

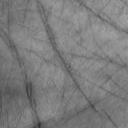

Supplement: S1 File — (ZIP) [file pone.0178432.s001.zip › Palmprint/B_0008_12.jpg]

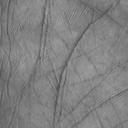

Supplement: S1 File — (ZIP) [file pone.0178432.s001.zip › Palmprint/B_0009_01.jpg]

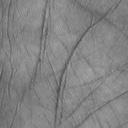

Supplement: S1 File — (ZIP) [file pone.0178432.s001.zip › Palmprint/B_0009_02.jpg]

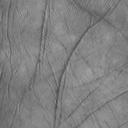

Supplement: S1 File — (ZIP) [file pone.0178432.s001.zip › Palmprint/B_0009_03.jpg]

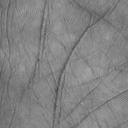

Supplement: S1 File — (ZIP) [file pone.0178432.s001.zip › Palmprint/B_0009_04.jpg]
